# Lysed erythrocyte membranes metabolically prime endothelial cells for angiogenesis

**DOI:** 10.1007/s10456-026-10096-y

**Published:** 2026-09-25

**Authors:** Rajinikanth Gogiraju, Kateryna Moiko, Magdalena L. Bochenek, Franziska Greulich, Claudius Witzler, Werner Schmitz, Konstantinos Zifkos, Cécile Derieux, Iman Ghasemi, Payal Guliani, Beichen Sun, Christine Espinola-Klein, Henriette N. Uhlenhaut, Andreas Bock, Wolfram Ruf, Thati Madhusudhan, Philipp Lurz, Katrin Schäfer

**Affiliations:** 1 Department of Cardiology, University Medical Center Mainz, Mainz, Germany; 2 German Center for Cardiovascular Research (DZHK E.V.), Site RheinMain (Mainz), Mainz, Germany; 3 Center for Thrombosis and Hemostasis, University Medical Center Mainz, Mainz, Germany; 4 Metabolic Programming, School of Life Sciences, ZIEL Institute for Food & Health, Technische Universität München, Freising, Germany; 5 Institute for Diabetes and Endocrinology Deutsches Zentrum Für Diabetesforschung, Helmholtz Munich, Neuherberg, Germany; 6 Institute of Biochemistry and Molecular Biology, University of Würzburg, Würzburg, Germany; 7 Institute of Pharmacology, University Medical Center Mainz, Mainz, Germany; 8 Department of Cardiology, Center for Translational Vascular Biology (CTVB), University Medical Center of the Johannes Gutenberg-University Mainz, Langenbeckstraße 1, 55131 Mainz, Germany; 9 Center for Immunotherapy (FZI), University Medical Center of the Johannes Gutenberg-University, Mainz, Germany

**Keywords:** Erythrocytes, Inflammation, Metabolism, Angiogenesis, Endothelial cells, Cardiovascular disease, Peripheral artery disease

## Abstract

Endothelial dysfunction is a hallmark of pathological angiogenesis and associated with erythrocyte extravasation and lysis. Whether mediators released from lysed erythrocytes instruct endothelial cells (ECs) for new vessel formation is unknown. Here, we show that the membrane fraction of lysed erythrocytes activates inflammatory endothelial gene transcription and metabolically reprograms ECs to promote angiogenic sprout formation. Alterations in endothelial glucose metabolism occurred downstream of NFκB p65 activation by extracellular ATP via fast-acting endothelial P2X7 receptors and also involved ATP-mediated adenylyl cyclase activation and cyclic AMP generation via P2RY11. Overexpression and acetylation of histone 3 at lysine 27 of NR4A1 was identified as anti-inflammatory control mechanism and angio-metabolic switch activating *VEGF*, *PFKFB3* and other AP-1 dependent gene transcription programs. The angiogenic potential of lysed erythrocyte membranes from patients with peripheral artery disease was impaired and could be restored by inhibiting endothelial phosphodiesterase 4 to prevent endothelial cAMP degradation. Our findings uncover that erythrolysis metabolically primes ECs for angiogenesis and that targeting endothelial cAMP generation may be a promising strategy to restore endothelial angiogenic functions.

**Supplementary Information:**

The online version contains supplementary material available at https://doi.org/10.1007/s10456-026-10096-y.

## Graphical abstract

This graph was created with BioRender.com (license agreement number: JP2A2V9VXQ).

**Figure:**
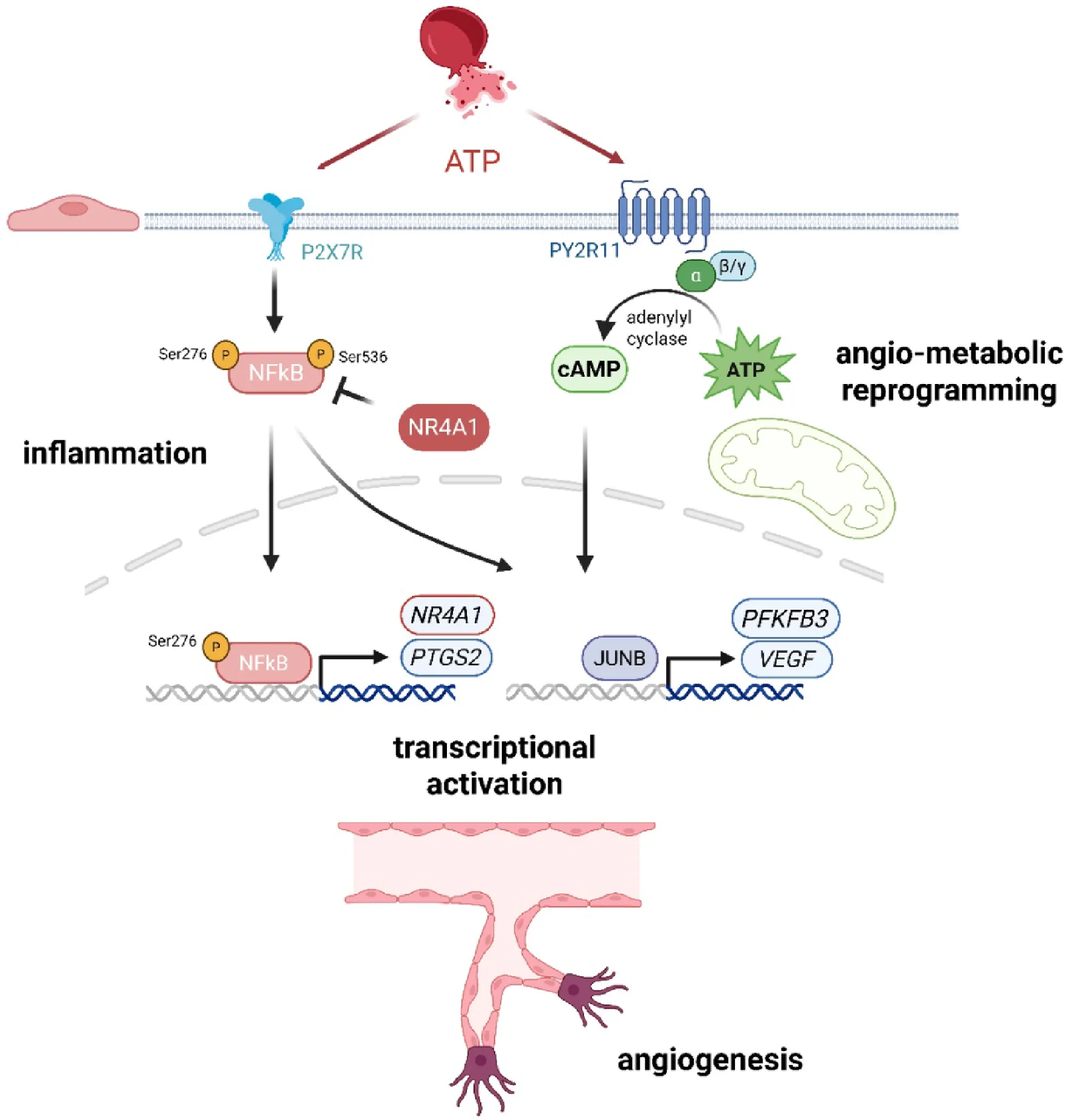

## Introduction

Endothelial cells (ECs) line the inner layer of all blood vessels and have central roles in vascular physiology maintaining endothelial barrier functions, controlling immune and platelet activation and ensuring proper tissue perfusion [1]. Following reductions in local blood flow and oxygen content, ECs first respond by the release of vasodilatory factors to maintain tissue perfusion [2, 3]. This early response is followed by endothelial gene expression changes towards angiogenic growth factors to build new blood vessels and to prevent ischemic tissue damage. In response to ischemia, but also other factors, quiescent endothelial cells rapidly switch their metabolism to generate the energy and biosynthetic precursors required for tip cell migration, stalk cell proliferation, and sprout cell elongation and differentiation [4]. On the other hand, the inability of ECs to respond in this manner and to build new blood vessels leads to defective angiogenesis, a hallmark of peripheral artery disease (PAD). The causes underlying the inability of ECs to proliferate, migrate and form tubes are multi-fold and include harmful effects of cardiovascular risk factors like age and diabetes mellitus promoting oxidative stress, inflammation and endothelial dysfunction [5], but also impact other cells regulating blood flow and tissue perfusion.

Erythrocytes (red blood cells, RBCs) are the most abundant cell type in circulating blood and constantly in direct contact with the vascular endothelium. The oxygen-dependent conformational change of hemoglobin to release vasoactive mediators constitutes a fundamental physiological mechanism of vascular tonus control [6]. In addition, erythrocytes express the enzymatic machinery necessary to synthesize high-energy substrates like adenosine 5’ triphosphate (ATP) [7], which is sequestered within the cytoskeletal-membrane complex [8]. From there, ATP can be released in response to reduced oxygen tension [9], but also mechanical deformation [10] or increased shear stress [11]. The local increase of ATP release from erythrocytes while passing through hypoxic or constricted blood vessels is a physiological mechanism to promote vasodilation thus increasing tissue perfusion and oxygenation. In addition to controlled RBC release, hemolysis is another important mechanism how RBCs release ATP [12, 13]. In fact, earlier studies suggested that hemolysis is the primary ATP release mechanism in human erythrocytes [12]. The lysis of erythrocytes may occur within blood vessels, for example following mechanical trauma on damaged endothelium, infections or autoimmune mechanisms, but also following erythrocyte leakage from structurally or functionally abnormal ‘pathological’ blood vessels, a hallmark of advanced cardiovascular disease [14]. Although hemorrhage and erythrocyte extravasation are frequently observed at sites of ischemia, they are mostly regarded as sequelae and late consequence of pathological angiogenesis, and not as its cause [14]. Whether factors like ATP released from lysed erythrocytes play active roles in the later phases of blood flow restoration by instructing ECs for new blood vessel formation has not been directly examined.

In this study, we hypothesized that erythrocytes, in particular factors other than hemoglobin set free during their lysis, modulate EC functions and actively contribute to the restoration of perfusion by promoting new blood vessel formation. Our findings demonstrate that the membrane fraction of lysed erythrocytes acts as ‘damage signal’ provoking an endothelial inflammatory response, but also reveal fundamental metabolic and transcriptional adaptations to promote endothelial proliferation, migration, sprout formation and tube elongation. Pharmacologically and genetically targeting endothelial ATP receptor signaling or the intracellular conversion of ATP to cAMP blunted the metabolic reprogramming and angiogenic phenotype conversion of ECs induced by RBC ghosts. Importantly, preventing cAMP degradation by inhibiting endothelial phosphodiesterase (PDE)− 4 restored the defective angiogenic response of ECs to lysed RBCs from patients with severe PAD. While these activities of RBCs lie dormant in an unperturbed, healthy state, they may represent an important regulatory mechanism in new vessel formation in the event of hemorrhage and erythrolysis, as frequently observed in cardiovascular disease, including arteriosclerosis and PAD.

## Results

### Lysed erythrocyte membranes activate the expression of genes associated with inflammation and angiogenesis in endothelial cells

To explore the effects of lysed RBCs on ECs, we performed bulk mRNA sequencing (RNA-seq). Human cardiac microvascular ECs were incubated with the hemoglobin-free, membrane fraction of RBCs (so-called ‘ghosts’), isolated from fresh (not stored) anticoagulated whole blood of healthy volunteers (Fig. S1A–C). ECs and RBC ghosts were incubated for two hours to detect early transcriptional changes and to minimize potential bias by secondary alterations. Flow cytometry analysis of CD41-positive cells excluded relevant platelet contamination (< 0.01%; not shown).

**Fig. 1:**
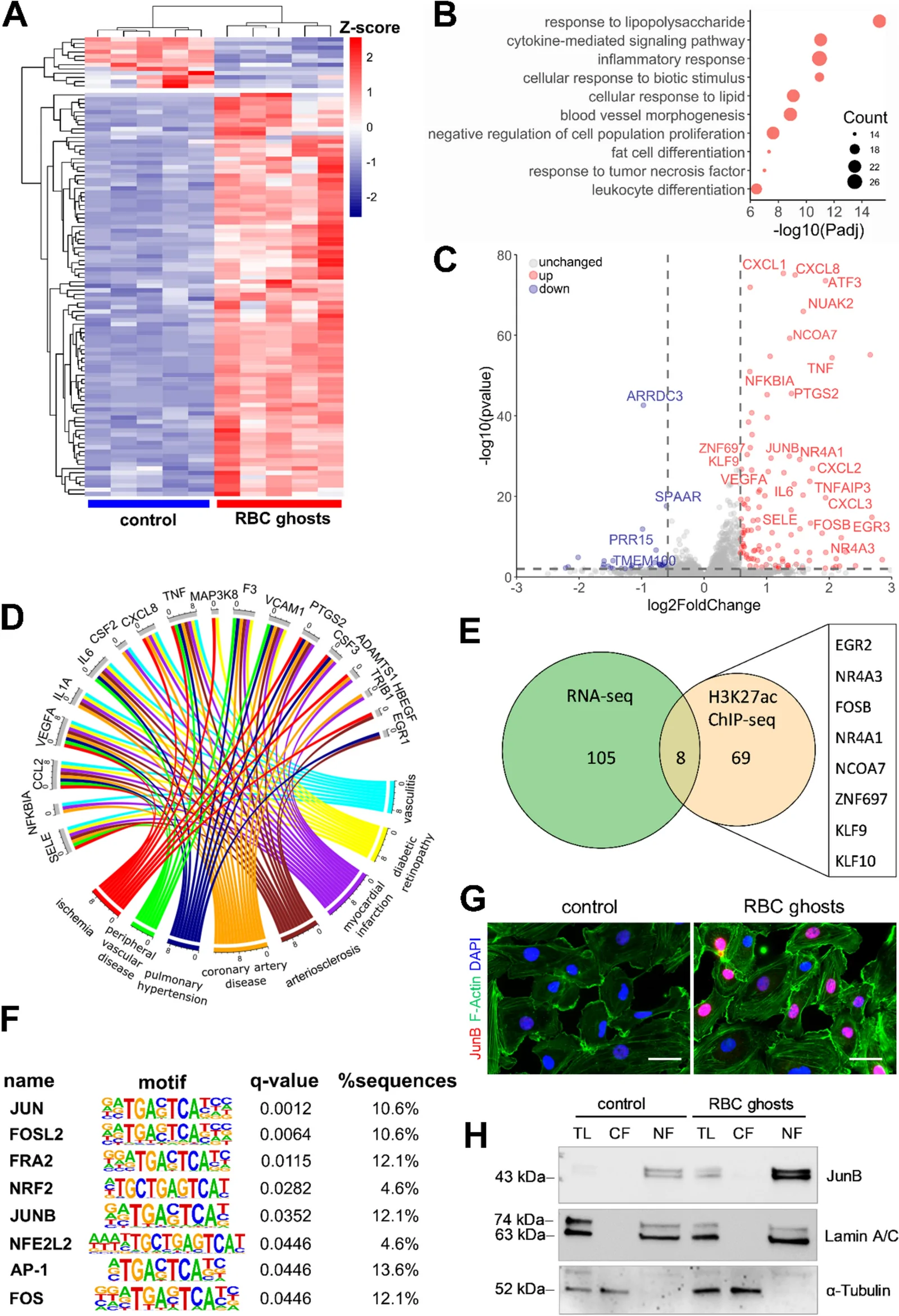
Lysed erythrocyte membranes activate the expression of genes associated with inflammation and angiogenesis in endothelial cells. **A** RNA-sequencing analysis of human cardiac microvascular ECs, untreated (control) and incubated with lysed erythrocyte membranes (RBC ghosts) for two hours. Heatmap showing all DEGs (absolute fold-change >1.5-fold, Benjamini-Hochberg-adjusted *P*-value <0.05). Color indicates Z-score. n=5 biologically independent samples (RBC donors) per condition. **B** Top ten over-represented gene ontology terms (GO) for biological processes of differential expressed genes (as in A). The node size reflects the gene count. **C** Volcano plot DEGs [− log10 (*P*-value)] versus magnitude of change (log2FoldChange. Selected up- (red) or down- (blue) regulated genes are labeled. **D** Chord diagram illustrating the relationships between selected DEGs (top) and enriched DO terms (bottom). The colored chords represent DO related to ischemia (red), peripheral vascular disease (green), pulmonary hypertension (blue), coronary artery disease (orange), arteriosclerosis (brown), myocardial infarction (purple), diabetic retinopathy (yellow), and vasculitis (turquoise). **E** Differentially expressed genes and differential H3K27 acetylation at 2 h-treatment with RBC ghosts in ECs plotted as venn diagram. Overlapping genes are shown in the box. **F** Homer result showing all significant motifs (q-value <0.05) including q-value and % of target sequences. **G** Immunocytochemical staining of JUNB in HCMECs, control and RBC ghost-treated for two hours. Nuclei are counter-stained by DAPI (blue). Scale bars, 50 µm. **H** Cellular fractionation and analysis of the cytoplasmic (validated using α-Tubulin as marker) and nuclear (validated using Lamin A/C as marker) localization of JUNB in control and RBC ghost-treated HCMECs. Representative immunoblot membrane is shown. Please note that the same membrane was used to detect NR4A1 nuclear translocation and that the Lamin A/C control band is also shown in Fig. 5F. The full gel image is provided in the supplements

Bioinformatic analyses revealed 113 differentially expressed genes (DEGs; 97 upregulated, 16 downregulated) in EC incubated with RBC ghosts with a false discovery rate (FDR) of 0.05 and a fold-change of at least 1.5 fold compared to untreated ECs (control) (Fig. 1A). Principal Component Analysis showed that RBC ghost-treated ECs clustered separately from controls in Principal Component 1 (PC1) explaining 23.2% of the observed variance (not shown). Among the top 15 gene ontology (GO) terms with the highest number of DEGs were ‘response to lipopolysaccharide’ and ‘inflammatory response’, but also ‘blood vessel morphogenesis’ (Fig. 1B). In addition to early growth response genes (*EGR2* and *EGR3*) and transcriptional regulators involved in the cellular response to stress (*ATF3*, *NUAK2*), mediators with known functions in inflammation and angiogenesis (*NFKBIA*, *PTGS2*, *TNFA, VEGF*) were among the highly significantly upregulated genes within these biological pathways (Fig. 1C). Bioinformatic analysis revealed DEGs in the disease pathways ‘ischemia’, ‘peripheral vascular disease’, ‘arteriosclerosis’, ‘myocardial infarction’, or ‘diabetic retinopathy’, among others (Fig. 1D).

To study whether the endothelial gene expression changes in response to RBC ghosts involve histone modifications, we performed chromatin immunoprecipitation followed by genome-wide high-throughput sequencing (ChIP-seq) analysis. Antibodies against histone 3 acetylation at lysine 27 (H3K27ac) were used as marker of active enhancers. A total of 77 differentially H3K27 acetylated sites (fold-change < 1.5-fold, *p*-value < 0.05) between RBC ghost treated and untreated samples were observed, of which 60 gained (85.7%) and 14 (14.3%) lost H3K27 acetylation. Integration of the epigenetic and transcriptome data showed 8 overlapping and 69 unique targets (Fig. 1E). Motif enrichment analysis of genomic regions with increased H3K27 acetylation revealed a predominance of members of the activator protein-1 (AP-1) family (Fig. 1F), activated by inflammatory cytokines and other cellular stressors, accounting for 55% (88 out of 160) of the target gene sequences. Immunofluorescence microscopy (Fig. 1G) and cellular fractionation (Fig. 1H) confirmed the increased presence of JUNB in the nuclear fraction of ECs exposed to RBC ghosts.

### Lysed erythrocyte membranes promote NFκB activation in endothelial cells

**Fig. 2:**
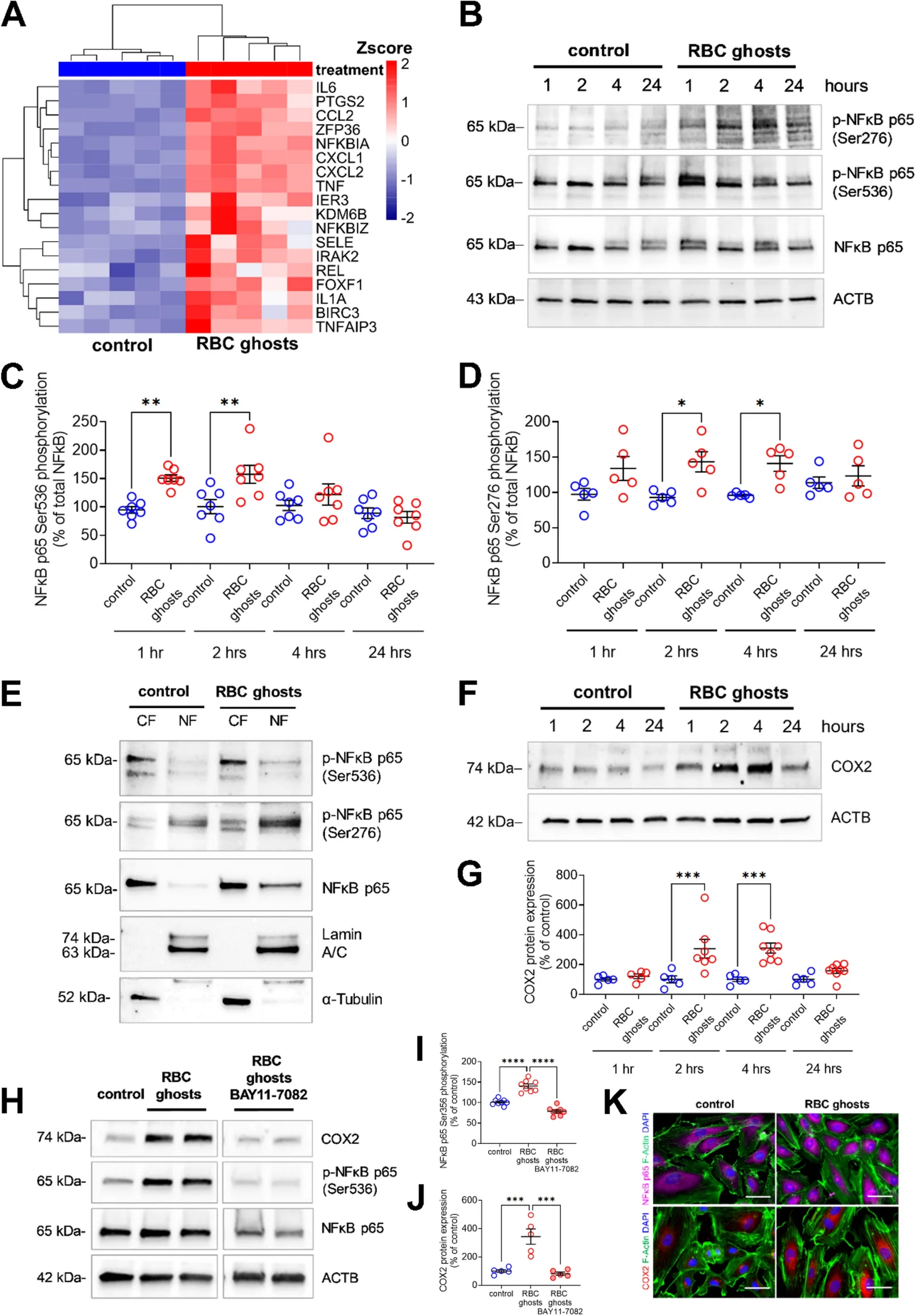
Lysed erythrocyte membranes promote NFκB activation and COX2 expression in endothelial cells. **A** Heatmaps of DEGs annotated with the GO term ‘inflammatory response’ (GO: 0006954). Color indicates Z-scores. **B-D** Immunoblot analysis of NFκB p65 subunit phosphorylation at Ser536 (B, C) and Ser276 (B, D) in response to RBC ghost exposure for the indicated time points. n=7 [C] and n=5 [D] biologically independent samples. *P < 0.05 and **P < 0.01 (one-way ANOVA, Sidak’s multiple comparisons test). **E** Cellular fractionation and localization of NFκB p65, total and phosphorylated at Ser536 or Ser276 in ECs, untreated (control) or incubated with RBC ghosts for two hours. A immunoblot membrane representative of two independent experiments is shown. Cytoplasmic fraction (CF) and nuclear fraction (NF). **F, G** Immunoblot analysis of COX2 protein expression in response to RBC ghost exposure for the indicated time points. n=5–8 biologically independent samples. ***P < 0.001 (one-way ANOVA, Sidak’s multiple comparisons test). **H-J** Effect of IκBα inhibitor BAY11-7082 (5 µM) along with RBC ghosts on NFκB p65 Ser536 phosphorylation (H, I) and COX2 (H, J) protein expression after 2-h incubation. n=5–8 biologically independent samples. ***P < 0.001 and ****P < 0.0001 (one-way ANOVA, Sidak’s multiple comparisons test). **K** Immunocytochemical detection of NFκB p65 (upper row) and COX2 (lower row) in ECs, untreated (control) or incubated with RBC ghosts for two hours. Scale bars, 50 µm

Our findings suggested that RBC ghosts promote the inflammatory activation of ECs. Hallmark genes associated with the GO term ‘inflammatory response’ (GO: 0006954) are shown in Fig. 2A and included members of the NF-kappa-B (NFκB) family as well as NFκB target genes (e.g., *CCL2*, *IL6*, *PTGS2*, *TNF*). Protein levels of the NFκB subunit p65, phosphorylated at serine−536 (Ser^536^), the site phosphorylated by multiple kinases downstream of inflammatory stimuli [15, 16], were significantly increased in ECs exposed to RBC ghosts for one and two hours (Fig. 2B, C). Increased NFκB p65 phosphorylation at Ser276, the site phosphorylated by cyclic AMP-dependent protein kinase (PKA) and critical for regulating its transcriptional activity [17], was observed later, after co-incubation of RBC ghosts with ECs for two and four hours (Fig. 2B, D). NFκB p65 phosphorylated at Ser276 was also the predominant form detected in the nuclear fraction of ECs stimulated with RBC ghosts (Fig. 2E). Protein levels of the NFκB-regulated inflammatory mediator COX2 (encoded by the *PTGS2* gene) also significantly increased after co-incubation for two and four hours (Fig. 2F, G). The IκBα inhibitor BAY11-7082 prevented the increase in NFκB Ser536 phosphorylation (Fig. 2H, I) and COX2 protein expression (Fig. 2H, J) induced by RBC ghosts, confirming NFκB p65 activation as central mediator of the pro-inflammatory expression changes observed in ECs exposed to RBC ghosts. Immunocytochemistry confirmed the elevated NFκB p65 (Fig. 2K, upper row) and COX2 (Fig. 2K, lower row) expression in ECs exposed to RBC ghosts. Thus, RBC ghosts induced NFκB-mediated inflammatory endothelial activation.

### Lysed erythrocytes promote endothelial proliferation, migration and sprout formation

**Fig. 3:**
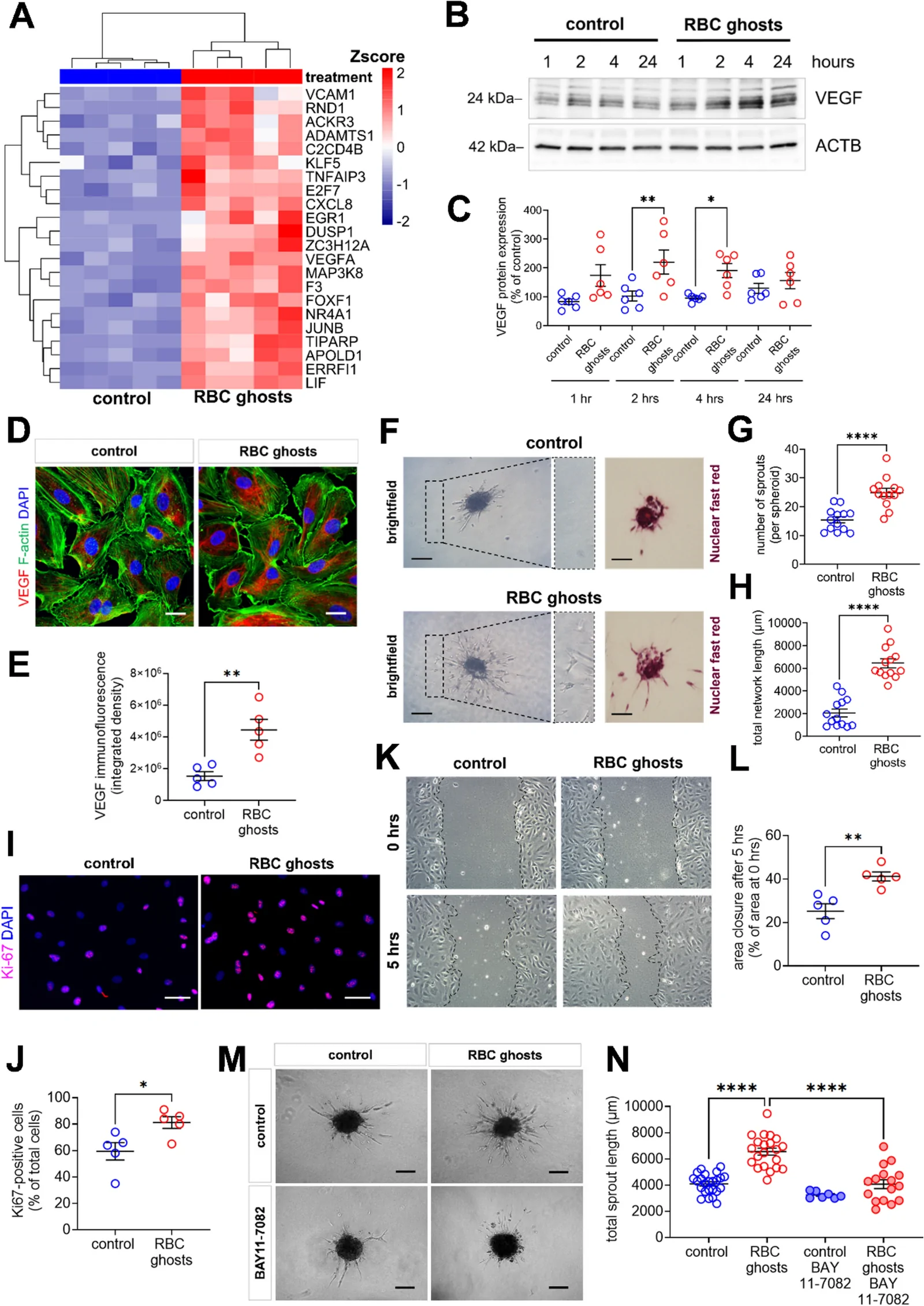
Lysed erythrocyte membranes promote endothelial sprouting and new blood vessel formation. **A** Heatmaps of DEGs annotated with the GO terms ‘vascular development’ (GO:0001944), ‘regulation of cell–cell adhesion’ (GO: 0022407) and ‘regulation of cell adhesion’ (GO:0030155). Color indicates Z-scores. **B, C** Immunoblot detection of VEGF protein expression in ECs, exposed to RBC ghosts for 1, 2, 4 and 24 h. Representative membrane (B). Quantitative analysis (C). n=6 biologically independent RBC samples. *P < 0.05, **P < 0.01 (one-way ANOVA, Sidak ‘s multiple comparisons test). **D, E** Immunofluorescence staining of VEGF in ECs, control or incubated with RBC ghosts for four hours. Representative confocal microscopy images (D). Quantitative analysis (E). Scale bars, 5 µm. Mean of four images in n=5 biologically independent samples (RBC donors) per condition. **P < 0.01 (Student ‘s t-test). **F–H** Spheroid angiogenesis assay. Representative images (F). Quantification of the number of sprouts per spheroid (G) and the total endothelial network length (H). Mean findings in at least ten spheroids per human RBC donor (n=13; 62% male, 24–56 years-of-age). Scale bars, 200 µm. ****P <0.0001 (Student’s t test). **I, J** Ki-67 immunofluorescence staining of proliferating ECs, control and after incubation with RBC ghosts for four hours. Representative images (I). Scale bars, 50 µm. Quantitative analysis (J). Mean of five images in n=5 biologically independent samples (RBC donors) collected per sample, per condition. *P < 0.05 (Student ‘s t-test). **K, L** Analysis of EC migration using the scratch wound assay. Representative images (K), and quantitative analysis of the relative wound closure after five hours (L). Mean of four images in n=5 biologically independent samples per condition. **P < 0.01 (Student ‘s t-test). MF, microscopic field. **M, N** Effect of the IκBα inhibitor BAY11-7082 (5 µM) on endothelial angiogenic sprout formation, examined in the spheroid angiogenesis assay. Representative findings are shown in (***N***), the quantitative analysis of the total endothelial network length in (O). Mean findings from n=3 RBC donors per condition, with multiple spheroids analyzed per condition for each donor. Each dot represents one individual spheroid. Scale bars, 200 µm. ****P < 0.0001 (one-way ANOVA, Sidak’s multiple comparison test)

Our transcriptome and bioinformatic analyses suggested that endothelial activation in response to RBC ghosts also contributed to new blood vessel formation. Hallmark genes associated with the GO terms ‘blood vessel morphogenesis’ (GO: 0048514), ‘regulation of cell–cell adhesion’ (GO: 0022407) or ‘positive regulation of cell migration’ (GO: 0030335) are shown in Fig. 3A. Among those, we selected the pro-angiogenic growth factor VEGF for further validation. Increased VEGF protein expression in ECs exposed to RBC ghosts for two hours and longer was confirmed at the protein level using immunoblot (Fig. 3B, C) and immunofluorescence microscopy (Fig. 3D, E). To determine the effects of RBC ghosts on endothelial angiogenic functions, we employed the spheroid angiogenesis assay. Co-incubation of ECs with RBC ghosts increased the number of sprouts (Fig. 3F, G) and the total network length (Fig. 3F, H) per endothelial spheroid, similar to recombinant human VEGF, used as positive control (Fig. S2A–C). Of note, VEGF protein was observed in ECs, but not in RBC ghosts (Fig. S2D). RBC ghosts did not alter mRNA transcript levels of VEGFRs (Fig. S2E) or protein expression of VEGFR2 (Fig. S2F, G). Incubation of ECs with RBC ghosts also significantly enhanced the number of Ki-67 positive, proliferating ECs (Fig. 3I, J) and promoted their migratory capacities in the scratch-wound assay (Fig. 3K, L). The endothelial sprout formation in response to RBC ghosts was abolished after incubation of ECs with BAY11-7082 (Fig. 3M, N).

Although total lysed erythrocytes (containing both the cytosolic and the membrane fraction) were also effective, the hemoglobin- and cytoplasm-free erythrocyte membrane preparation (so-called ‘ghosts’) was sufficient to promote endothelial sprouting and tubular network formation (Fig. S3A–C). Similar findings were observed using the Matrigel™ angiogenesis assay (Fig. S3D–F). Heme isolated from whole blood preparations did not affect angiogenic sprout formation (Fig. S4A, B). Perl’s iron stain showed no or only discrete staining signals indicative of iron or its metabolites in ECs incubated with RBC ghosts (Fig. S4C). Thus, RBC ghosts but not heme promoted endothelial cell angiogenic responses.

### Lysed erythrocyte membranes promote sprouting angiogenesis by reprogramming endothelial metabolism

**Fig. 4:**
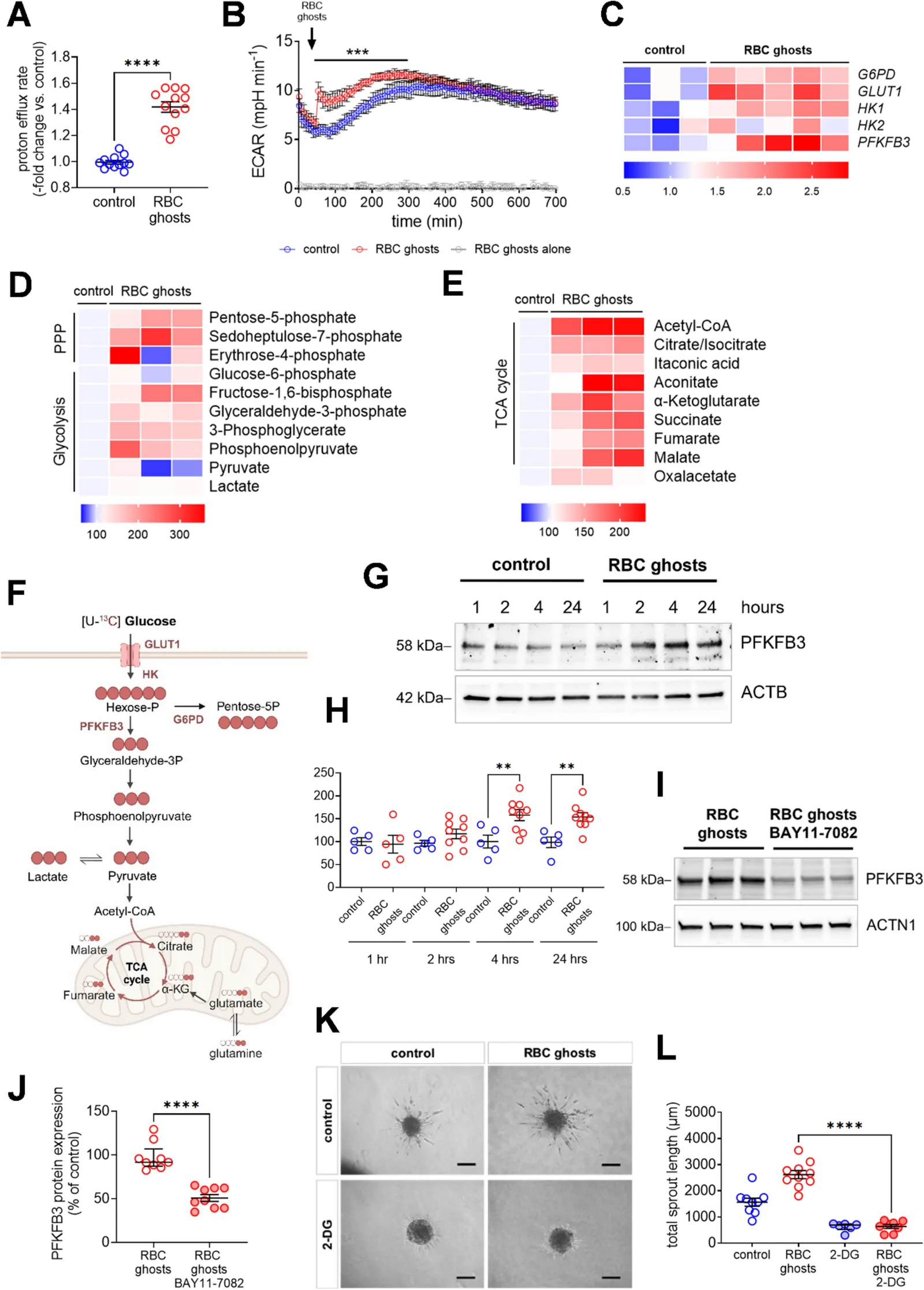
Lysed erythrocyte membranes promote sprouting angiogenesis by reprogramming endothelial metabolism. **A** Calculated proton efflux rate (PER). ****P < 0.0001 (Student’s t test). **B** Real time measurement of the extracellular acidification rate (ECAR) in ECs incubated with RBC ghosts compared to untreated control ECs measured using Seahorse (n=3–6 biological replicates, representative of n=3 independent experiments). ***P < 0.001 (two-way ANOVA, Sidak’s multiple comparisons test). **C** Heatmap showing quantitative PCR analysis of mRNA expression of glycolytic markers in ECs exposed to RBC ghosts for 2 h (n=3 control and n=5 RBC ghost-treated samples). **D, E** Metabolomics analysis using LS/MS. Determination of metabolite levels in glycolysis and the pentose phosphate pathway (D) and acetyl-CoA levels and TCA cycle metabolites (E) in cellular extracts of ECs, untreated (control) or incubated with RBC ghosts for two hours. Peak areas were normalized to the internal standard (Lamivudine) and are expressed relative to untreated ECs (control). Data for RBC ghosts treated ECs are shown as the average normalized peak area (% of control). **F **Schematic summary of metabolic labeling. The image was created using BioRender.com. **G, H** Immunoblot showing PFKFB3 protein levels in ECs incubated with RBC ghosts for 1, 2, 4 and 24 h. Representative membrane (G). Quantitative analysis (H). n=5–9 biologically independent RBC samples. **P < 0.01 (one-way ANOVA, Sidak’s multiple comparisons test). **I, J** PFKFB3 protein levels in ECs, incubated with RBC ghosts for two hours, alone or in the presence of the IκBα inhibitor BAY11-7082 (5 µM). Representative immunoblot membrane (I). Quantitative analysis (J). n=5–9 biologically independent RBC samples. ****P <0.0001 (Mann–Whitney test). **K, L** Sprouting angiogenesis of human endothelial cells incubated with RBC ghosts, control or in the presence of the non-metabolizable glucose analog 2-DG (5 mM), examined in the spheroid angiogenesis assay. Representative images (K) and quantitative analysis of total endothelial network length (L). Mean findings from n=2 RBC donors per condition, with multiple spheroids analyzed per condition for each donor. Each dot represents one individual spheroid. Scale bars, 200 µm. ****P <0.0001 (Kruskall-Wallis, Dunn’s multiple comparisons test)

The generation of energy by converting glucose to pyruvate (glycolysis) is a critical event in ECs during new blood vessel formation [4]. Agilent Seahorse XF analysis showed that adding RBC ghosts to endothelial monolayers significantly enhanced the proton efflux rate (PER; Fig. 4A) and extracellular acidification rate (ECAR; Fig. 4B). The RBC ghost-induced increase in endothelial glycolysis occurred within minutes and was sustained for approximately five hours after which values returned to those in untreated ECs. No detectable metabolic changes were observed in RBC ghosts alone under these experimental conditions, suggesting that the metabolic alterations are due to endothelial responses to RBC ghost-derived signals and not to an intrinsic metabolic activity of the ghost fraction itself. Quantitative real time PCR analysis confirmed significantly higher mRNA levels of glucose transporter type 1 (*GLUT1*) and the key glycolytic enzymes hexokinase 1 (*HK1*), *HK2*, glucose−6-phosphate dehydrogenase (*G6PD*) and *PFKFB3* (6-phosphofructo-2-kinase/fructose−2,6-biphosphatase 3) in ECs exposed to RBC ghosts for 2 h (Fig. 4C). PKFB3 has been identified in previous studies as key metabolic hub regulating the metabolic reprogramming during angiogenesis [18], and was therefore selected for further validation.

LS/MS based metabolomics confirmed increased levels of glycolysis intermediates and lower levels of pyruvate in ECs incubated with RBC ghosts compared to controls (Fig. 4D). These analyses also revealed increased pentose phosphate pathway intermediates, suggesting that RBC ghosts activate anabolic side branches of glycolysis. Metabolic flux analysis confirmed the increased generation of biosynthetic precursors in ECs incubated with RBC ghosts, and also showed their absence in RBC ghosts alone (Fig. S5A–C). Incubation of ECs with RBC ghosts also increased the production of acetyl-CoA and intermediates of the TCA cycle (Fig. 4E). The results of the metabolic flux analysis are summarized in Fig. 4F.

Linking the effects of RBCs on endothelial metabolism with their angio-inductive properties, significantly increased protein levels of endothelial PFKFB3 after co-incubation with RBC ghosts for four and 24 h were confirmed by immunoblot (Fig. 4G, H). Previous work established the central role of PFKFB3 in metabolically reprogramming ECs during vessel sprouting and angiogenesis [18]. Importantly, inhibiting NFκB activation with BAY11-7082 significantly reduced *PFKFB3* mRNA (not shown) and protein levels (Fig. 4I, J) in ECs treated with RBC ghosts. The non-metabolizable glucose analogue 2-deoxy-D-glucose (2-DG) also abolished the endothelial sprout formation in response to RBC ghosts (Fig. 4K, L).

Mitochondrial ATP production in ECs did not change until approximately 11 h after the addition of RBC ghosts (Fig. S6A). A significantly increased spare respiratory capacity and higher oxygen consumption rate (OCR) was observed in RBC ghost-treated ECs after injection of the electron transport chain uncoupling agent FCCP (carbonyl cyanide p-trifluoromethoxyphenyl hydrazone; Fig. S6B, C), unmasking the ability of cells to respond to increased energy demands or stress. Whereas neither UK-5099 to inhibit the mitochondrial pyruvate carrier (Fig. S7A, B) nor etomoxir to inhibit carnitine palmitoyltransferase 1a (Fig. S7C, D) prevented the increased endothelial sprouting activities in response to RBC ghosts, inhibiting the glutamine oxidation pathway using BPTES (Bis-2-(5-phenylacetamido-1,3,4-thiadiazol-2-yl) ethyl sulfide) reduced the RBC ghost-induced endothelial sprout formation to levels seen in control cells (Fig. S7E, F). These data suggest that RBC ghosts promote glutaminolysis to support mitochondrial anaplerosis by endothelial production of α-ketoglutarate to replenish the TCA cycle. Tracing experiments using U-^13^C labeled glucose showed that ribose−5P routed into purine synthesis at two hours, while U-^13^C labeled glutamine confirmed enhanced influx and conversion into glutamate for pyrimidine synthesis at six hours (not shown). These data support an anabolic profile in RBC ghost-treated ECs with time-dependent, variable demands for building blocks during endothelial sprout formation.

### NR4A1 mediates the endothelial metabolic and angiogenic changes in response to lysed erythrocyte membranes

**Fig. 5:**
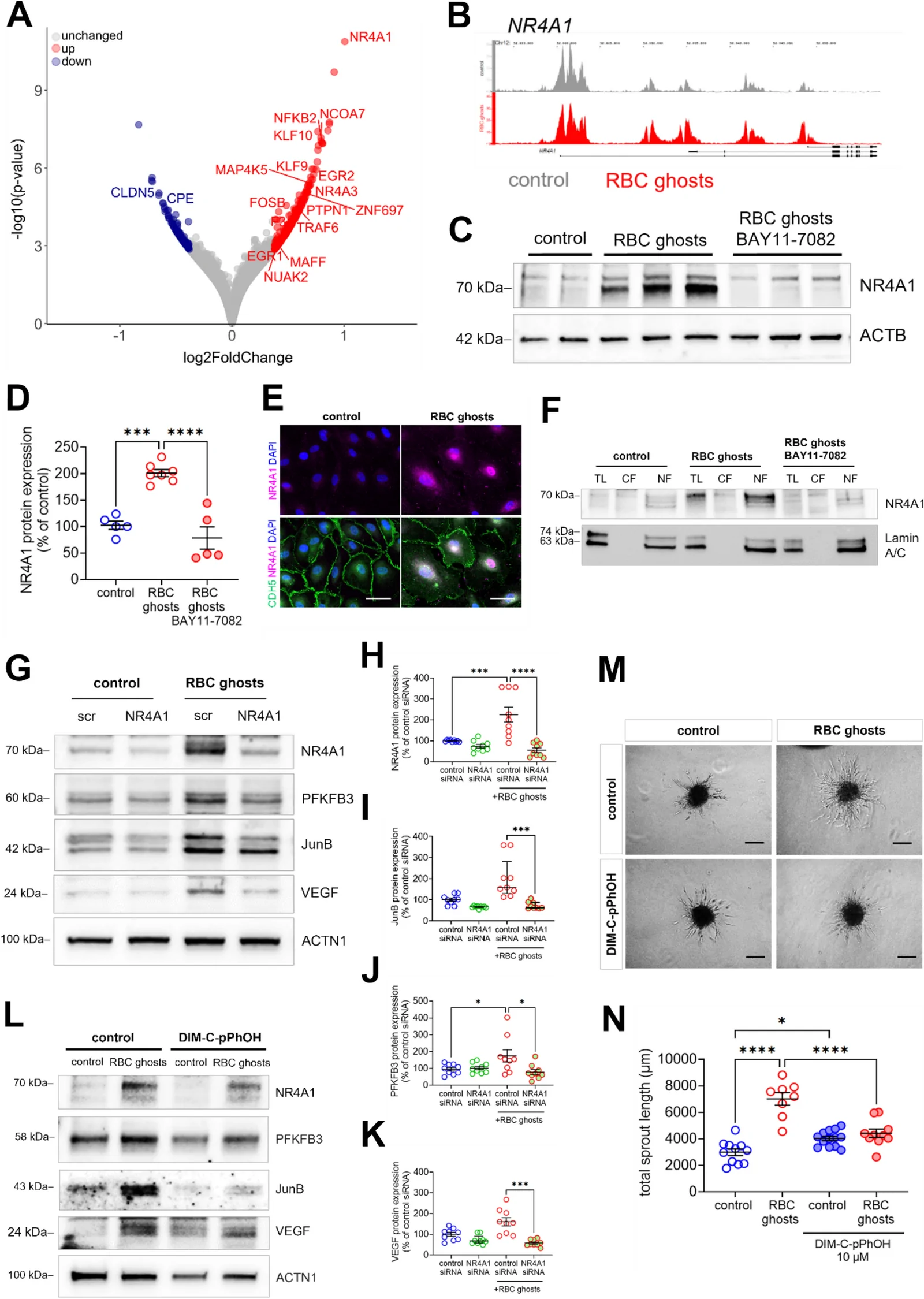
Overexpression and nuclear translocation of NR4A1 mediates the endothelial metabolic and angiogenic changes in response to lysed erythrocyte membranes. **A **Volcano plot of genes associated with significantly (P<0.05, fold-change >1.3-fold) gained (red) or lost (blue) lost H3K27acetylation. **B **H3K27ac ChIP seq tracks for genomic regions at the *NR4A1* gene. **C, D** NR4A1 protein expression in ECs, untreated (controls) or exposed to lysed erythrocyte membranes (RBC ghosts) for two hours, alone or in the presence of the IκBα inhibitor BAY11-7082 (5 µM). Representative immunoblot membrane (C). Quantitative analysis (D) (n=5–6 biologically independent RBC samples). ***P <0.001 and ****P <0.0001 (one-way ANOVA, Sidak’s multiple comparisons test). **E** Immunofluorescence staining of NR4A1 in ECs, untreated (control) or incubated with RBC ghosts for two hours. Scale bars, 50 µm. CDH5 immunostaining was used to visualize endothelial tight junctions. **F** Cellular fractionation followed by immunoblot analysis of NR4A1 in ECs, TL: total lysate, CF: cytoplasmic fraction, NF: nuclear fraction (validated using Lamin A/C as marker). The same membrane was used for JUNB detection (shown in Fig. 1H). **G-L** Effect NR4A1 downregulation (using siRNA; G-K) on the expression of NR4A1 (H) JUNB (I), PFKFB3 (J) and VEGF (K) protein (n=3 biologically independent RBC samples with three technical repeats) or antagonization (using 10 µM DIM-C-pPhOH; **L**). Representative immunoblot membranes (G, L). Quantitative analysis (H–K). *P <0.05, ***P <0.001 and ****P <0.0001 (one-way ANOVA, Sidak’s multiple comparisons test in H and J; Kruskall-Wallis, Dunn’s multiple comparisons test in I and K). **M, N** Sprouting angiogenesis of ECs incubated with RBC ghosts, control or in the presence of the NR4A1 antagonist DIM-C-pPhOH (10 µM), examined in the spheroid angiogenesis assay. Representative images (M). Quantitative analysis of the total endothelial network length (N). Mean findings from n=2 RBC donors per condition, with multiple spheroids analyzed per condition for each donor. Each dot represents one individual spheroid. Scale bars, 200 µm. *P <0.05 and ****P <0.0001 (one-way ANOVA, Sidak’s multiple comparisons test)

We next sought to identify the NFκB dependent transcriptional modulator driving the metabolic and angiogenic changes in ECs in response to RBC ghosts. Our RNA-seq and H3K27ac ChIP-seq analyses uncovered Nuclear Receptor Subfamily 4 Group A (*NR4A1*, also known as nuclear hormone receptor NUR77), as one of the genes most strongly and significantly upregulated by RBC ghosts (data shown in Fig. 1C). NR4A3 was also detected in the transcriptomic dataset but the degree and consistency of induction was more pronounced for NR4A1, leading to its prioritization for further mechanistic analysis. A Volcano plot showing genes with differential H3K27 acetylation is displayed in Fig. 5A, genome browser tracks of H3K27ac at *NR4A1* in Fig. 5B. Previous studies have demonstrated roles for NR4A1 during angiogenesis [19], metabolism [20] and inflammation [21, 22]. Immunoblot analysis confirmed strongly increased NR4A1 protein expression in ECs incubated with RBC ghosts for two hours, whereas NR4A1 protein levels were low and almost undetectable in control cells (Fig. 5C, D). Inhibiting NFκB activation with BAY11-7082 abolished the RBC ghost-induced increase in endothelial NR4A1 protein expression, in line with two highly conserved NFκB response elements in the NR4A1 promoter [21]. Fluorescence immunocytochemistry (Fig. 5E) and cellular fractionation (Fig. 5F) showed the predominant presence of NR4A1 in the nuclear fraction of ECs exposed to RBC ghosts and its dependence on NFκB activation.

Previous work established that NR4A1 can activate or repress gene expression by assisting other transcription factors [23], including JUNB and others within the AP-1 family [24]. Importantly, siRNA-mediated NR4A1 downregulation (Fig. 5G, H) prevented the RBC ghost-induced increase in endothelial protein levels of JUNB (Fig. 5G, I), PFKFB3 (Fig. 5G, J) and VEGF (Fig. 5G, K), supporting its role in the transcriptional control of these metabolic and angiogenic genes. Similar findings were observed applying a NR4A1 antagonist (Fig. 5L). Inhibiting NR4A1 activation significantly reduced endothelial sprout formation in response to RBC ghosts (Fig. 5M, N). Notably, protein levels of the immediate early gene NR4A1 were only transiently upregulated at two hours and thereafter returned to baseline levels (Fig. S8A, B), and siRNA-mediated knockdown of NR4A1 significantly increased endothelial NFκB p65 phosphorylation already in the absence of RBC ghosts (Fig. S8C, D). These data show that NR4A1 is regulated by NFκB, but also part of a negative feedback loop to limit endothelial inflammation, while reprogramming metabolism and promoting angiogenesis.

### Endothelial NR4A1 overexpression occurs in response to ATP

**Fig. 6:**
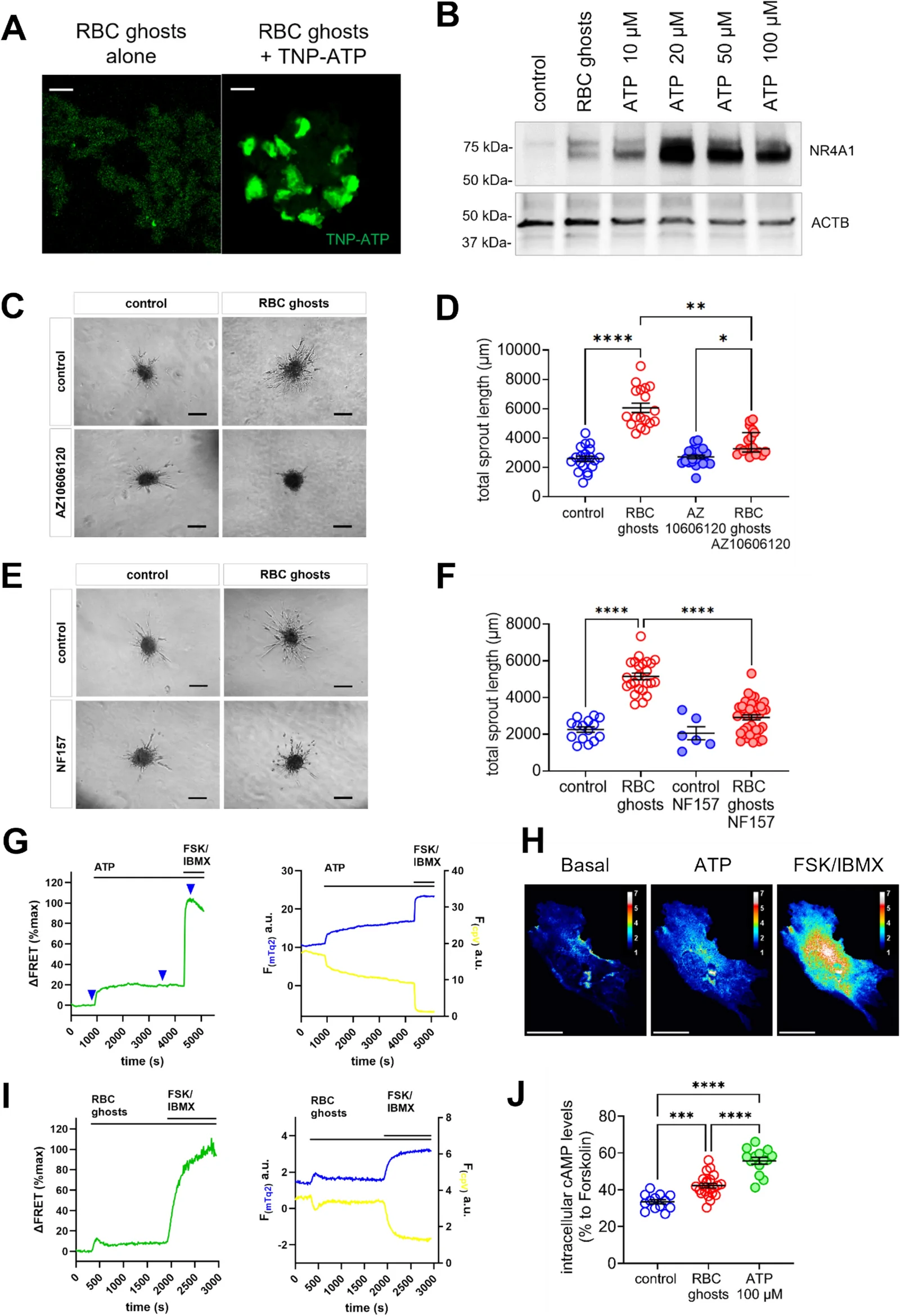
Endothelial NR4A1 overexpression occurs in response to ATP in lysed erythrocyte membranes. **A** Labeling of ATP- binding sites in human RBC membranes using the fluorescent ATP analogue TNP-ATP (green, upper panel) and negative control without TNP-ATP (lower panel). Scale bars, 5 µm. **B** Changes in NR4A1 protein expression in response to RBC ghosts and extracellular ATP (10, 20, 50 and 100 µM) for two hours. Immunoblot membrane representative of two experiments. **C-F** Effect of ATP P2X7 receptor inhibition (using AZ10606120) or P2RY11 inhibition (using NF157) on endothelial sprout formation in response to RBC ghosts. Mean findings from n=3 RBC donors per condition, with multiple spheroids analyzed per condition for each donor. Each dot represents one individual spheroid. Scale bars, 200 µm. *P <0.05, **P <0.01, and ****P <0.0001 (D: Kruskall-Wallis, Dunn’s multiple comparisons test; F: one-way ANOVA, Sidak’s multiple comparisons test). **G** Stimulation of endogenous purine receptors with ATP (100 µM) results in a robust and stable cAMP increase. *Left*, representative trace of corrected and normalized FRET ratios in a single endothelial cell (EC) transiently expressing the FRET-based Epac-S^H187^ cAMP-biosensor, recorded by single-cell FRET-imaging. The trace is normalized to baseline (set to 0%) and to FSK (10 µM) / IBMX (100 µM) (i.e.’max’, set to 100%). *Right*, corresponding single fluorescence intensity traces (mTurquoise2 (FRET-donor), blue; cpVenus (FRET-acceptor), yellow). **H** Pseudocolored FRET images (scale bar, 50 µm) corresponding to the experiment in (G) illustrate cAMP production (color bar, increasing cAMP concentration with increasing ratio values) at different time points (depicted as blue arrows in the left panel in (G)). **I** Under identical imaging conditions, stimulation with RBC ghosts elicits a more transient but robust cAMP response similar to ATP. *Left*, representative trace of corrected and normalized FRET ratios in a single EC. *Right*, corresponding single fluorescence intensity traces. Data in (G) and (I) are representative examples from three and two independent experiments, respectively. **J** Whole-cell cAMP levels measured by HTRF confirm cAMP production in response to RBC ghosts and ATP (100 µM). n=3–4 biologically independent samples, examined in three independent experiments. ***P <0.001 and ****P <0.0001, (one-way ANOVA with Sidak’s multiple comparisons test)

We focused our next analyses on identifying the causal factor in the membrane fraction of erythrocytes that triggers endothelial NR4A1 expression. Because the endogenous ligands activating this orphan nuclear receptor have not yet been identified, we reasoned that ATP, stored in RBC membrane microdomains [8] and released during hemolysis [12, 13], may have mediated the inflammatory endothelial activation and upregulation of NR4A1. Labeling of RBCs with the fluorescent ATP analogue TNP (2’,3’-O-Trinitrophenyl)-ATP (Fig. 6A) and flow cytometry analysis using ATP-Red, a cell-permeable ATP sensor (Fig. S9A, B) confirmed the presence of ATP binding sites or functional ATP in RBC membranes. Platelets, a circulating cell type known to store ATP, were used as positive control in the latter assay. Endothelial protein levels of NR4A1 strongly increased in response to stimulation with high (i.e. 10 µM and higher) concentrations of ATP, present at sites of inflammation [25] or released from RBCs in hypoxic microvessels [26], to levels similar to those in RBC ghost-treated ECs (Fig. 6B). Incubation of ECs with ATP (100 µM) promoted endothelial sprout formation (Fig. S9C, D) and tube elongation (Fig. S9C, E) similar to RBC ghosts. ATP also recapitulated the RBC ghost-induced increase in *RELA* (Fig. S9F), *PFKFB3* (Fig. S9G) and *VEGF* (Fig. S9H) mRNA transcript levels, thus confirming the importance of ATP for the activation of the key components of the angio-metabolic reprogramming downstream of RBC ghosts identified in our study. Conversely, pretreatment of RBC ghosts with apyrase to degrade extracellular ATP significantly reduced the RBC ghost-induced angiogenic sprout formation (Fig. S10A, B) and endothelial expression of NR4A1, PFKFB3 and VEGF (Fig. S10C). We interpreted the observation that apyrase did not completely prevent the pro-angiogenic effects of RBC ghosts as indirect evidence that they trigger intracellular changes in endothelial cells not amenable to extracellular ATP degradation by apyrase, including metabolic ATP generation.

### Lysed erythrocyte membranes activate endothelial P2X7 as well as P2Y11 receptors coupling to adenylyl cyclase activation and cAMP generation

Further experiments were performed to determine the signaling cascade elicited by extracellular ATP. Blocking endothelial ATP signaling via P2X7 receptors, nucleotide-gated ion channels mediating the pro-inflammatory effects of extracellular ATP [25], using the allosteric P2X7R antagonist AZ10606120 significantly reduced the angiogenic effects of RBC ghosts, albeit not completely (Fig. 6C, D). ATP also activates metabotropic P2Y receptors, G-protein-coupled receptors that mediate slower, modulatory effects compared to fast-acting ionotropic P2X7Rs [2]. RNA-seq detected gene transcripts for P2RY1, 2, 6 and 11 in ECs, control and RBC ghost-treated, out of which P2RY11 expression levels were highest (Fig. S11A). Blocking endothelial ATP signaling using the selective P2RY11 antagonist NF157 abolished endothelial angiogenic sprouting in response to RBC ghosts (Fig. 6E, F). Findings using pharmacological inhibitors were confirmed in ECs transfected with siRNA targeting P2X7R or P2Y11R (Fig. S11B–D). From these findings we concluded that the activation of P2X7 and P2Y11 receptors on ECs by ATP present in the RBC ghosts is necessary to convert the pro-inflammatory trigger into a sustained proangiogenic response.

To visualize changes in endothelial cAMP levels in response to ATP RBC ghosts, HCMECs were transfected with genetically encoded, fluorescence resonance energy transfer (FRET)-based cAMP biosensor, i.e. Epac-S^H187^ [27]. Real-time, single-cell FRET imaging demonstrated that addition of ATP (Fig. 6G, H) and RBC ghosts (Fig. 6I) to intact HCMECs resulted in significant increase in cytosolic cAMP concentrations. These cAMP responses were very robust albeit smaller than the maximal cAMP response upon adenylyl cyclase (AC) activation (forskolin) and phosphodiesterase (PDE) inhibition (3-isobutyl-1-methylxanthine, IBMX). Quantitative analysis of total intracellular cAMP accumulation over two hours using an HTRF-based assay confirmed the whole-cell cAMP increase in response to ATP and RBC ghosts (Fig. 6J). Findings that endothelial angiogenic sprout formation significantly increased in response to the cell-permeable cAMP analogue 8-bromo-cAMP (Fig. S12A) or pharmacological approaches inhibiting cAMP degradation, such as the non-selective PDE inhibitor IBMX (Fig. S12B) or rolipram to specifically inhibit PDE4, a cAMP-specific PDE highly expressed in ECs (Fig. S12C), further supported a role for cAMP in mediating the angiogenic response to RBC ghosts. Using forskolin to activate membrane-bound AC (mAC) associated with stimulatory GPCRs phenocopied the effects of RBC ghosts on endothelial sprout formation (Fig. S13A), whereas HCO_3-_ to activate soluble AC was ineffective (Fig. S13B). Those observations are in line with that SQ22536, a cell-permeable mAC inhibitor without effects on sAC [28], prevented angiogenic sprout formation induced by RBC ghosts (Fig. S13C, D). SQ22536 also reduced the RBC ghost-induced endothelial NFκB p65 phosphorylation at Ser276 and protein expression of JUNB and VEGF (Fig. S13E). From these data we concluded that endothelial cAMP generation via P2Y11 (and possibly also other) receptors coupling to mAC is necessary to fully activate NFκB p65 and to promote AP-1 dependent gene transcription and angiogenesis.

### The angio-metabolic reprogramming is blunted in endothelial cells exposed to lysed erythrocyte membranes from patients with severe peripheral artery disease

**Fig. 7:**
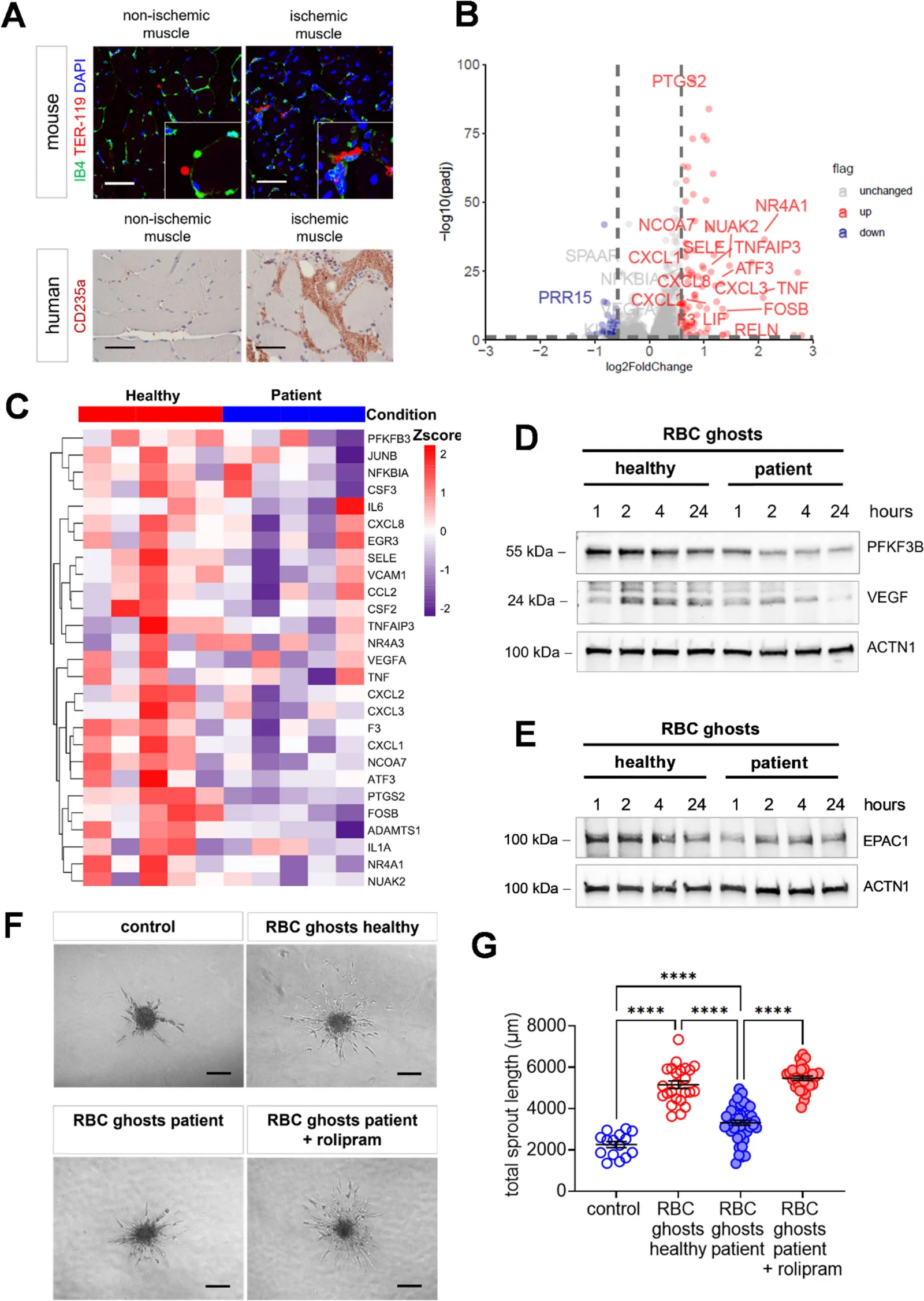
The angio-metabolic reprogramming is blunted in endothelial cells exposed to lysed erythrocyte membranes from patients with severe peripheral artery disease. **A** Immunolabeling of murine (TER-119 positive) and human (CD235a positive) erythrocytes in ischemic (mouse: day 14 after induction of hindlimb ischemia; human: peripheral artery disease [PAD], stage III/IV) and non-ischemic skeletal muscle tissue. **B** Volcano plot showing DEGs in ECs treated with RBC ghostsfrom patients with PAD, stage III/IV (n=5; patient) and age-matched control subjects without known cardiovascular risk factors or disease (n=5; healthy). **C** Heatmap showing differences between ECs exposed to RBC ghosts from n=5 healthy controls or n=5 PAD patients for 2 h in the expression of central mediators and DEGs involved in ‘inflammation’, ‘angiogenesis’ and ‘metabolism’. Color indicates Z-scores. **D, E** Immunoblot membrane showing changes of PFKFB3, VEGF (D) and Exchange Protein Activated by cAMP 1 (EPAC1) (E) protein levels in ECs incubated with RBC ghosts from a healthy donor or an age- and sex-matched patient PAD for 1, 2, 4 and 24 h. **F, G** Spheroid angiogenesis assay to evaluate the angiogenic properties of RBC ghosts from healthy donors and patients, alone or in the endothelial PDE4 inhibitor rolipram (10 µM). Representative images (F) and quantification of the total network length (G). Mean findings from n=3 RBC donors per condition, with multiple spheroids analyzed per condition for each donor. Each dot represents one individual spheroid. Scale bars, 200 µm. ****P < 0.0001 (one-way ANOVA, Sidak’s multiple comparisons test)

Impaired neovascularization and defective vascular regeneration are hallmark features of PAD. Hemorrhage and erythrocyte extravasation are often observed at sites of ischemia, as confirmed by immunofluorescence staining of ischemic skeletal muscle tissue from C57BL/6 J mice and patients with severe ischemic PAD (Fig. 7A). We therefore selected PAD as a clinically relevant disease setting and asked whether RBC ghosts from PAD patients that is, persons with clinically defective angiogenesis, continue to induce metabolic and angiogenic expression changes in ECs. We hypothesized that the pro-angiogenic activities of RBC ghosts from patients with PAD may be compromised in comparison to RBC ghosts from age- and sex-matched donors without cardiovascular risk factors (‘healthy’). RNA-seq analysis revealed the upregulation of pro-inflammatory mediators in ECs exposed to RBC ghosts, independent of whether they were isolated from PAD patients and healthy persons without known cardiovascular risk factors or disease (Fig. 7B). However, direct comparison of mRNA transcript levels of the central mediators of metabolic reprogramming and angiogenesis identified in our study revealed significantly lower expression levels in ECs stimulated with RBC ghosts from patients compared to those stimulated with RBC ghosts from healthy controls (Fig. 7C). The blunted upregulation of PFKFB3 and VEGF (Fig. 7D), but also of Exchange Protein Directly Activated by cAMP 1 (EPAC1) in ECs treated with patient RBC ghosts was confirmed on the protein level (Fig. 7E). In line, their pro-angiogenic activities, although present, were significantly lower compared to RBC ghosts from healthy controls (Fig. 7F, G). Importantly, pharmacologically increasing endothelial cAMP levels by inhibiting PDE4 and thus cAMP hydrolysis restored the angiogenic response to levels seen in response to healthy RBC ghosts (Fig. 7F, G).

Collectively, our findings show that RBC ghosts act as damage signals to metabolically prime ECs for increased proliferation, migration and angiogenic sprout formation. Extracellular ATP acting on endothelial P2X7 receptors was identified as the triggering, priming event to promote inflammatory activation, and endothelial cAMP generation downstream of P2RY11 as the mechanism involved in endothelial transcriptional reprogramming to fully enable them for angiogenesis. The significance and clinical implications of our data for vascular disease are underlined by findings that the angiogenic response to RBC ghosts from patients with PAD was blunted and could be restored by inhibiting endothelial cAMP degradation.

## Discussion

The formation of new blood vessels is vital to prevent ischemic tissue injury, and both insufficient and excessive angiogenesis are involved in disease. Here, we show that the hemoglobin-free membrane fraction of lysed erythrocytes actively promotes endothelial proliferation, migration and angiogenic sprout formation. The proangiogenic effects of erythrocyte membranes occurred downstream of endothelial NFκB activation and involved profound changes in inflammatory and angiogenic gene transcription as well as metabolic reprogramming. Upregulation of NR4A1 was identified as a central regulatory node and angio-metabolic switch downstream of ATP and NFκB, acting as negative feedback mechanism to prevent endothelial inflammatory overaction while promoting AP-1 controlled transcription of angiogenic and metabolic genes. We identified ATP in RBC membrane microdomains as causal agent and show that endothelial sprout formation occurs via P2X7R, but also P2RY11 to induce cAMP generation to sustain the angio-metabolic endothelial alterations and enabling cAMP response element binding protein (CREB) and AP-1 mediated gene transcription changes. Validation studies in age- and sex-matched PAD patients and controls was used to test the translational relevance of the angiogenic mechanism identified in vitro and to determine if changes in RBC ATP-dependent signaling are linked to impaired angiogenic activity in a disease with defective vascular regeneration. Currently, our understanding of angiogenesis is centered on endothelial and mesenchymal cells as the principal cellular players during new blood vessel formation, with roles for immune cells and activated platelets in the setting of inflammation or thrombosis. Our findings add hemorrhage and erythrocyte lysis as an important initiating event in neovascularization and suggest that ATP stored in the erythrocyte membrane and released during their lysis promotes endothelial cell adaptations necessary for new vessel formation. Importantly, the angiogenic endothelial response to RBC ghosts from PAD patients and upregulation of EPAC1, PFKFB3 and VEGF was blunted, but could be restored by preventing endothelial cAMP degradation.

Increased vascular permeability, erythrocyte extravasation and lysis are commonly seen at the site of inflammation and ischemia. Our epigenetic and transcriptome sequencing analyses uncovered that RBC ghosts induce endothelial inflammatory activation and that inhibiting NFκB activation not only prevented the endothelial activation, but also the metabolic reprogramming and angiogenesis. Hemolysis is a known mechanism of endothelial inflammatory activation [29]. Autoxidation of hemoglobin leading to the release of superoxide from erythrocytes was proposed earlier as a mechanism of endothelial NFκB activation and pro-inflammatory gene transcription in hypoxic lung inflammation [30]. Here, we used the hemoglobin-free (‘white’) membrane fraction of lysed erythrocytes and show that it is sufficient to induce the inflammatory metabolic activation in ECs and to promote angiogenesis. We also directly excluded a role for heme, giving rise to the initial ferric superoxide intermediate [31], as mediator of the angiogenic activities of RBC ghosts.

The bioenergetic profiling and metabolic flux analyses in our study show that RBC ghosts promote angiogenesis by increasing endothelial glycolysis and glutaminolysis and diverting glycolytic metabolites to the pentose phosphate pathway. These metabolic alterations are typical features of highly proliferative cells, and our data suggest that RBC ghosts actively induce these metabolic adaptations to support ECs during new vessel formation. The rapid increase in endothelial proton flux following the addition of RBC ghosts suggests an initial membrane translocation of pre-existing glucose transporters, in line with previous work in immune cells following Toll-like receptor agonist stimulation [32]. Metabolic reprogramming of ECs and activation of the NFκB–PFKFB3 axis in response to lipopolysaccharide, TNFα and other pro-inflammatory mediators was reported earlier [33]. Our findings put these data into a pathophysiologically relevant context by showing that ‘metabolic inflammation’ occurs in response to the membrane fraction of lysed erythrocytes and promotes endothelial functions related to angiogenesis. Importantly, preventing NFκB activation abolished the endothelial PFKFB3 overexpression and angiogenic sprout formation induced by RBC ghosts, positioning it as the initial trigger and priming event. Metabolic epigenetic modifications and activating histone acetylations may sustain the RBC ghost-induced alterations in ECs.

NR4A1 was identified as one of the most prominently upregulated genes in response to RBC ghosts, and experiments using NR4A1 siRNA or antagonists confirmed its crucial role in promoting the nuclear translocation of JUNB, a member of the immediate-early transcription factor AP-1 family identified in our ChIP-seq analyses. High JUNB expression is specifically seen in tip cells at the angiogenic front of developing blood vessels [34]. Other members of the NR4A family of nuclear receptors also were upregulated in response to RBC ghosts, such as NR4A3, a hypoxia-inducible factor regulating glucose uptake [35]. NR4A receptors are ligand-*in*dependent transcriptional regulators integrating cellular metabolism [20], angiogenesis [19] and vascular remodeling [36] downstream of growth factors, inflammatory mediators and other stressors. Fitting the time scale of early inflammatory endothelial activation and importance of hemolysis as the primary ATP release mechanism from RBCs [12], ATP and NFκB activation were identified as upstream regulator of endothelial NR4A1 expression in our study. Of note, NR4A1 overexpression was transient, peaking at two hours, and NR4A1 downregulation or inhibition prevented the metabolic and angiogenic changes, while further increasing the inflammatory alterations, corroborating earlier reports in immune cells [22, 37]. This observation suggests that the upregulation of NR4A1 in ECs in response to RBC ghosts not only mediates the transcriptional reprogramming, but also constitutes a negative feedback loop to prevent endothelial inflammatory overactivation and damage. Of note, our study was not designed to systematically explore the relative contributions of all NR4A family members. NR4A receptors are structurally homologous, recognize similar DNA response elements, and have been reported to have both overlapping and context-dependent functions in vascular biology. Therefore, we cannot exclude that NR4A3 or other NR4A family members contribute to selected aspects of the endothelial response described herein. Future studies will be required to determine the relative contributions of NR4A1 and other NR4A family members in the endothelial response to RBC ghosts.

How do lysed erythrocytes promote early endothelial metabolic inflammation? One of the best studied, universal damage-associated molecular pattern molecule is extracellular ATP [38]. While present at negligible concentrations in the extracellular environment under physiological conditions, high levels of extracellular ATP accumulate during inflammation, hypoxia or cellular damage [39]. Mechanical deformation [11] or hemolysis [12, 13] are other major mechanism of ATP release from the RBC membrane. The accumulation of erythrocyte membranes at the site of hemorrhage and erythrolysis thus very likely results in the deposition of large quantities of extracellular ATP. Although extracellular nucleotides released from activated platelets or dying cells may act in a similar fashion and are present at sites of erythrocyte extravasation and lysis in the clinical scenario, they were carefully removed during the RBC ghost preparation [40]. Proangiogenic effects of extracellular ATP were already suggested by findings that ATP stimulates endothelial mitosis and migration and the expansion of the vasa vasorum network in the pulmonary artery [41]. However, the cellular and molecular events underlying this observation were largely unknown.

Proangiogenic effects in our study were seen in response to ATP concentrations required for signaling via P2X7R receptors to activate NFκB [39, 42], but also to activate the adenylyl cyclase activity of P2Y11 receptors to increase cAMP generation [43]. Pharmacological P2X7R and P2Y11R inhibition and siRNA-mediated genetic deletion blunted the endothelial response to RBC ghosts, supporting P2X7 and P2Y11 as the major components of the response although contributions of other purinergic receptors or receptors coupling to cAMP generation cannot be excluded. The importance of cAMP generation is highlighted by our findings of an impaired angiogenic response of ECs to patient RBC ghosts in parallel with reduced EPAC1, PFKFB3 and VEGF expression and the efficacy of PDE4 inhibition in restoring endothelial sprout formation. Although our findings in patients should be considered exploratory due to the small sample size and do not to infer clinical efficacy of PDE4 inhibition, the clinical benefits of PDE3 inhibitors like cilostazol in improving surrogate markers of ischemia in patients with intermittent claudication has already been documented in clinical trials [44]. Our data uncover the importance of this pathway for endothelial metabolic activities linked to angiogenesis. Of note, PDE3 inhibitors were not taken by the PAD patients of whom RBCs were isolated for analysis, and one out of five patients was taking P2Y12 inhibitors. Nevertheless, we cannot exclude that risk factors or medication may have affected our findings in PAD patients, and further studies in larger cohorts of age- and sex-matched patient and control samples are necessary to fully substantiate our findings. In addition to acting via purinergic receptors, extracellular ATP may elevate intracellular cAMP levels by sensitizing AC and enhancing its response to other agonists acting at G_S_-coupled receptors, such as prostaglandin E1 or epinephrine [45]. Potential ligands activating endothelial cAMP generation via auto- or paracrine activation of stimulatory G_S_ proteins in our study include prostaglandins, generated by activated ECs in response to RBC ghosts via COX2 upregulation. Notably, the 8-base palindrome 5'-TGACGTCA-3', identified in our ChIP-seq analysis, is also the binding site for CREB (cAMP Response Element-Binding protein) [46], and cAMP response elements are present in the promoter region of COX2 [47] and PFKFB3 [48].

## Conclusion

Our findings reveal that RBC ghosts trigger inflammatory and metabolic endothelial adaptations to promote angiogenesis and that the mechanisms involve NFκB activation by extracellular ATP and P2X7 receptors, NR4A1 overexpression and AP-1 mediated transcriptional changes inducing metabolic reprogramming. We also show that extracellular ATP signaling via endothelial P2Y11 receptors induces a more sustained endothelial response and that the activation of cAMP generation via membrane-bound adenylyl cyclase is necessary to fully rewire inflammatory endothelial activation into a reparative, proangiogenic response. We acknowledge the fact that the time dependence of intracellular signaling events and cross-talks is difficult to disentangle and that pharmacological inhibitor or siRNA deletion approaches will give hints, but do not allow to established temporal hierarchies beyond a certain time point and after the initial trigger. From our data, we conclude that RBC ghosts sustain endothelial pro-angiogenic activation through complementary and mutually reinforcing mechanisms. Also, while ATP was identified as major mediator and necessary for the observed response, this does not exclude contributions from other RBC membrane-associated factors. Since P2Y11 receptors are not expressed in mice, experimental validation using an animal model of ischemia was not feasible, and we acknowledge this important limitation. Instead, and to determine the translational relevance of the ATP–P2Y11–cAMP signaling axis identified in our study, we examined RBC ghosts from PAD patients. Our findings that the pro-angiogenic capacities of RBC ghosts were lost in patients with clinically confirmed defective angiogenesis and that they could be rescued by preventing cAMP degradation further support the mechanistic framework discovered in this study. Conceptually, ATP, but also other factors released from lysed erythrocytes may support EC functions under conditions of hypoxic stress to promote new vessel formation. Likewise, inhibiting endothelial inflammatory angio-metabolic signaling may serve as a therapeutic strategy in disease states where erythrocyte extravasation and blood vessel formation frequently coexist and are undesirable, such as the pathological angiogenesis seen in cardiovascular disease, cancer or specific forms of eye disease. Targeting metabolic pathways in ECs is being explored as therapeutic option to modulate new vessel formation [49], and our data advocate for taking the effects of lysed erythrocytes on endothelial metabolic inflammation into consideration in these disease scenarios.

## Methods

All unique/stable materials and protocols generated in this study are available from corresponding author without restriction, but requiring reasonable compensation by requestor for its processing and shipping or a completed Materials Transfer Agreement if there is potential for commercial application. All of the data that support the findings of this study are included in the article. Any additional information required to reanalyze the data reported in this paper is available from the corresponding author contact upon request.

A table listing key resources is provided as separate file.

### Erythrocyte isolation and fractionation

Human erythrocytes were isolated from anticoagulated whole blood of healthy human volunteers (n=9 biological replicates; 54% male; 39.2±3.0 years-of-age). In some experiments (data in Fig. 7), RBCs from patients with peripheral artery disease stage III/IV (n=5, 60% male, mean age: 61.2±2.1 years-of-age; 60% smoker, 60% diabetes mellitus, 40% dyslipidemia) were examined and compared to age- and sex-matched control persons without known cardiovascular risk factors or disease (‘healthy’; n=5, mean age: 64.6±3.4 years-of-age), described in more detail in [50]. The study was conducted in accordance with the Declaration of Helsinki, and approved by the Ethics Committee of the State Medical Association of Rhineland-Palatinate, Germany (Landesärztekammer Rheinland-Pfalz; approval number: 2019–14205-Klinische Forschung, approval date: 03 May 2019). Wrtten informed consent was obtained from all participants prior to inclusion in the study. Human erythrocytes were lysed to remove hemoglobin and cytoplasmic material and their membrane fraction was isolated, as described [40]. In brief, whole blood was collected in sterile vacutainers containing 3.2% citrate (S-Monovette® Citrate 9NC 0.106 mol L^−1^, Sarstedt, 04.1922) and centrifuged at 1,500 × g for 10 min to separate the plasma. The pellet was subjected to density gradient centrifugation (700 × g for 30 min) over Histopaque®-1119 (Sigma, 11,191) to remove leucocytes and platelets. Intact erythrocytes were lysed in hypotonic buffer (1 mM Tris–HCl, 1 mM EDTA, and 10 mM NaCl, pH 7.2) for 60 min at 4 °C to generate hemoglobin-free erythrocyte membranes (‘ghosts’). The membrane fraction was washed three times (13,000 × g for 15 min at 4 °C) in ice-cold 1X phosphate-buffered saline (PBS; Gibco, 14,190–094). Platelets were isolated from citrated human blood by sequential centrifugation to obtain platelet-rich plasma (PRP) and lysed using the above described protocol to obtain platelet membranes. Total protein content of the RBC membrane pellet was estimated using the Pierce™ BCA Protein Assay Kit (Thermo Scientific, 23,225), and 50 µg mL^−1^ of RBC membrane protein was used in all assays. Heme was isolated from 1 mL of whole blood using the acetone and ethyl acetate-acetic acid extraction method, as described [40]. In brief, for 1 mL of whole blood, ten volumes of acetone were added for at least 30 min at − 20 °C to promote heme precipitation followed by centrifugation at 1,500 × g for 10 min. The heme pellet was purified using a combination of ethyl acetate and acetic acid (3:1). The organic phase, containing purified heme, was isolated from the aqueous phase through centrifugation (2,500 × g for 10 min). The heme pellet was washed with 10 mL of dH_2_O and dissolved in 500 µL of 0.1 M Na_2_CO_3_.

### Endothelial cell culture

HCMECs [PromoCell, C-12285; isolated from a 55-year-old and 58-year-old male. caucasian (lot number: 439Z034.7 and 511Z015.1) and a 57-year-old female, caucasian (lot number: 492Z009.4)] were cultured on 0.2% gelatin-coated tissue culture grade plates (Greiner Bio-One, 664,160) in endothelial cell basal medium MV2 (PromoCell, C-22221) containing supplement mix (PromoCell, C-39221, containing 0.5 ng/mL recombinant human VEGF 165). Media had been tested for the absence of microbial contaminants (fungi, bacteria, mycoplasma) by the manufacturer. At 80–90% confluency, RBC ghosts were added and co-incubated for different time points, as indicated in the text, alone or in the presence of the glycolysis inhibitor 2-DG (2-deoxy-D-glucose; 5 mM, Sigma, D8375), the glutaminase inhibitor II (BPTES; 15 µM; Sigma-Aldrich, 5.30030), the carnitine palmitoyltransferase inhibitor etomoxir (100 µM; Sigma-Aldrich, 236,020), the mitochondrial pyruvate transport inhibitor UK-5099 (20 µM; Sigma-Aldrich, PZ0160), the IκBα inhibitor BAY11-7082 (5 µM; Sigma-Aldrich, B5556), the NR4A1 antagonist DIM-C-pPhOH (10 µM; MedChemExpress, HY-112055), the GPR4 antagonist NE 52-QQ57 (2 µM; MedChemExpress, HY-101784), the adenylyl cyclase inhibitor SQ22536 (1 µM; MedChemExpress, HY-100396), 8-bromo-cAMP (100 µM; MedChemExpress, HY-12306A), adenosine 5-triphosphate (range of 10–100 µM; Sigma, A6419), apyrase (5 U mL^−1^; Sigma-Aldrich, A6535), the cAMP activator forskolin (5 µM; Cayman Chemical, 11,018), the PDE4 inhibitor rolipram (10 µM; MedChemExpress, HY-16900), the non-selective PDE inhibitor IBMX (100 µM; Sigma-Aldrich, I5879), the purinoceptor P2X7 negative allosteric modulator AZ10606120 dihydrochloride (10 µM; Sigma-Aldrich, SML3600), the purinergic receptor P2Y11 antagonist NF157 (10 µM; TOCRIS, 2450) or an equal volume of vehicle (dimethylsulfoxide, DMSO; [Applichem, A3672] or sterile saline [Braun]) alone. HCMECs were transfected with 100 pmol of human NR4A1 siRNA (sc-36109), P2X7 siRNA (sc-42575), or P2Y11 siRNA (sc-76026) or non-targeting 20–25 nt scrambled siRNA as a negative control, (sc-37007; all Santa Cruz Biotechnology) using Lipofectamine® RNAiMAX transfection reagent (Thermo Fisher Scientific, 13,778,075) and analysed 48 h later.

### RNA isolation and bulk RNA sequencing

Total RNA was extracted from HCMECs using the RNeasy Plus Mini Kit (Qiagen). In brief, cells were cultured in 10 cm culture dishes until subconfluency and treated with RBC ghosts isolated from healthy human volunteers (n=5 biologically independent samples; 60% male, 24–53 years-of-age) for two hours or left untreated (control; n=5 experimental repeats). The quality and quantity of isolated RNA was verified using spectrophotometry (Nanodrop; Thermo Scientific) and Agilent 2100 Bioanalyzer with the RNA 6000 Nano kit, following the manufacturer’s instructions. Library preparation and rRNA depletion were conducted using the TruSeq unstranded mRNA Library Prep kit (Illumina) starting with 1 µg of RNA for each biological triplicate. The samples were sequenced on the Illumina NovaSeq6000.

### Chromatin-Immunoprecipitation (ChIP) sequencing

Ten million HCMECs were used for each ChIP. The cells were fixed with 1% formaldehyde (methanol-free) for 10 min at room temperature and snap frozen. The IP was performed using 4 µg of rabbit anti-H3K27ac (abcam, ab4729, RRID:AB_2118291) using the ChIP-IT High Sensitivity kit (Active Motif, 53,040), according to manufacture’s instructions. The DNA was quantified via Qubit, and the enrichment was validated by qPCR. Libraries were generated with the Kappa Hyperprep kit (Roche), according to the manufacturer’s instructions, and sequenced on an Illumina NovaSeq6000.

### RNA- and ChIP-sequencing analysis

NGS data quality was assessed with FastQC (RRID: SCR 014583, http://www.bioinformatics.babraham.ac.uk/projects/fastqc/). For RNA-sequencing, gene-level quantification was performed with Salmon version 1.9.0 (RRID: SCR_017036). Settings were: -libType A, -gcBias, -biasSpeedSamp 5 using the hg38 (Relase 33, GRCh38.p13, hg38) reference transcriptome provided by Gencode. Gene count normalization and differential expression analysis were performed with DESeq2 version 1.40.2 (RRID: SCR_015687) after import of gene-level estimates with “tximport” version 1.28.0 (RRID: SCR_016752) in R (RRID: SCR_001905, R version 4.3.1).

For gene annotation, “AnnotationHub” version 3.8.0 (RRID: SCR_0024227), and genome information provided by Ensembl (GRCh38.p13) was used. Genes with at least 10 read counts, fold-change of 1.5-fold and Benjamini-Hochberg-adjusted p value <0.05 were called significantly changed. Plots were generated with “ggplot2” version 3.5.1 (RRID: SCR_014601), “pheatmap” version 1.0.12 (RRID: SCR_016418, https://github.com/raivokolde/pheatmap) or “circlize” version 0.4.16 (RRID: SCR_002141) packages. DO and GO enrichment was performed with “clusterProfiler” version 4.8.3 (RRID: SCR 016884) on the differentially expressed genes after RBC ghost treatment (absolute fold-change > 1.5-fold, Benjamini–Hochberg adjusted P value < 0.05). Heatmaps of genes significantly regulated by RBC ghost treatment (absolute fold-change > 1.5-fold, Benjamini-Hochberg-adjusted *P*-value < 0.05) and enriched in the indicated biological processes. RNA-seq data is available on the Gene Expression Omnibus (GEO) with the accession number GSE GSE329930.

ChIP-seq paired-end reads were mapped to the human reference genome hg38 (Ensembl GRCh38.p13) with BWA-MEM version 0.7.13 (RRID: SCR 010910), and PCR duplicates were removed using Picard Tools version 2.0.1 (RRID: SCR − 006525, http://picard.sourceforge.net/). For visualization, bam files were filtered for properly paired and mapped reads, and multimappers were removed with Samtools version 1.18 (RRID: SCR 002105). Alignments were converted to bigwig files, merging 10 bp per bin using ‘bamCoverage’ from the Deeptools package version 3.5.6 (RRID: SCR − 016366). Tracks were visualised with the Integrative Genome Browser (IGB). Peaks were called with MACS version 3.0.0a5 in BAMPE mode and an FDR cutoff of 0.05 over matched input controls. Blacklisted regions (https://github.com/Boyle-Lab/Blacklist/blob/master/lists/hg38-blacklist.v2.bed.gz) were removed from analyses. Peak annotation was performed in R version 4.1.1 (RRID: SCR 014601) using the ChIPpeakAnno package version 3.34.1 and annotation data from the Ensembl genome GRCh38.p13 (hg38).

The peak union of all replicates was used to determine reads in peaks (RiP) ratios and scaling factors to normalize for library size and background-to-noise ratio. Genome browser tracks were normalized by the RiP fraction and the mean tracks were generated with Deeptools version 3.5.6. Peaks were annotated to the closest gene expressed in HCMECs in any of the conditions with the ‘ChIPpeakAnno’ package version 3.34.1 (RRID: SCR 012828). Genes were called expressed when passing a mean expression value of the 25th percentile. Motif enrichment was performed on differentially acetylated peaks using HOMER version 4.11. ChIP-seq data is available on the Gene Expression Omnibus (GEO) with the accession number GSE GSE329932.

### RNA isolation and quantitative real-time PCR

Total RNA was extracted from HCMECs using TRI Reagent (Ambion). The concentration and purity of isolated RNA molecules were measured using spectrophotometry (Nanodrop; Thermo Scientific). One microgram of total RNA was subjected to treatment with DNase I (1 U µg^−1^ of total RNA; Sigma) and used to synthesize first strand cDNA with M-MLV Reverse Transcriptase (Promega). Quantitative real-time PCR (qPCR) was performed using SYBR Green (BioRad), the data were collected on the CFX Connect Real-Time PCR Detection System (BioRad). Melting temperature analysis was performed to confirm the specificity of the qRT-PCR reaction. The human oligonucleotide primers and their sequences are shown in Table S1. The qRT-PCR results were calculated using the delta-delta Ct (2−ΔΔCt) method. ΔCt was calculated by subtracting Ct values of β-actin or hypoxanthine phosphoribosyl transferase 1 (used as housekeeping genes) from the Ct values of the gene of interest. ΔΔCt was calculated with controls of the same experimental setup. Results are reported as -fold change relative to the mean of untreated cells.

### Protein isolation and immunoblot analysis

HCMECs were lysed in RIPA buffer containing phenylmethylsulfonylfluorid (PMSF; Cell Signaling Technology, 8553) and protease inhibitors (Thermo Scientific, 78,440). Protein lysates (35 µg) were separated by SDS-PAGE and transferred to nitrocellulose membranes (0.45 µm; Amersham Protran™, 10,600,002). After blocking with 5% bovine serum albumine (BSA; Roth, 2834), membranes were probed with primary antibodies against COX2 (Cell Signaling Technology, 12,282, RRID:AB_2571729), JUNB (Cell Signaling Technology, 3753, RRID:AB_2130002), NFκB p65 (Cell Signaling Technology, 6956, RRID:AB_10828935), phospho-NFκB p65, Ser536 (Cell Signaling Technology, 3033, RRID:AB_331284), phospho-NFκB p65 Ser276 (Novus Biologicals, NBP100-82086, RRID: AB 1144568), NR4A1 (Santa Cruz Biotechnology, 365,113, RRID:AB_10709310 and Cell Signaling Technology, 3960, RRID:AB_2153738), PFKFB3 (Proteintech, 13,763–1-AP, RRID:AB_2162854) or VEGF (Santa Cruz Biotechnology, 7269, RRID:AB_628430). Protein loading was normalized using mouse monoclonal ATCB antibody (abcam, ab8226, RRID:AB_306371) or rabbit polyclonal ACTN1 antibody (Cell Signaling Technology, 3134, RRID:AB_2223798). HRP-conjugated secondary antibodies (cytiva, Amersham™) and chemiluminescence substrate (Thermo Scientific, 34,580) were used for visualization. Densitometry analysis was performed with AlphaEaseFC 4.0 software. Data are normalized to the protein loading control and are expressed as -fold change of findings in control cells. Images of uncropped gels of all immunoblots shown in this study are provided as separate file.

### Cytoplasmic and nuclear fractionation

Total cell lysates containing both soluble cytoplasmic and nuclear proteins were separated stepwise using the NE-PER™ Nuclear and Cytoplasmic Extraction Reagents (Thermo Scientific, 78,833) and protease inhibitors (Thermo Scientific, 78,440), according to the manufacturer’s instructions. To assess the purity of cytoplasmic and nuclear cellular fractions, antibodies against lamin A/C (clone 4C11; Cell Signaling Technology, 4777, RRID:AB_10545756) and α-tubulin (Cell Signaling Technology, 2144, RRID:AB_2210548) were used.

### LC–MS metabolomic analysis

HCMECs were cultured on 0.2% gelatin-coated tissue culture grade plates (BD Falcon) and maintained in endothelial cell growth medium MV2 with supplement mix (PromoCell). At 80–90% confluency, the medium was replaced with Agilent Seahorse XF RPMI medium containing unlabeled glucose or [U-^13^C]-glucose (25 mM) in the presence or absence of RBC ghosts for metabolite quantification and metabolic flux analysis (MFA). C^13^ tracing started by replacing medium with glucose-free medium supplemented with 25 mM [U-^13^C]-glucose. After two hours of incubation in the presence of [U-^13^C]-glucose, cells were washed twice with ice cold 154 mM NH_4_OAc and scraped off in 500 µL MeOH/H_2_O (80/20 v/v). Cell suspensions (n=3) or 10 µL culture media in 500 µL MeOH/H_2_O (80/20) (n=3) were spiked with 5 pmol lamivudine as internal standard and treated with ultrasound (5*0.5 s at max. output energy (Branson Sonifier 250 / cup horn, Emerson Electric Co., Saint Louis, USA)). After mixing and centrifugation (2 min at max. rpm in an Eppendorf centrifuge), the resulting supernatants were transferred to activated (by flushing with 500 µL CH_3_CN) and equilibrated (by flushing with 500 µL MeOH/H_2_O (80/20, v/v)) SPE columns (Strata C18-E, 50 mg mL^−1^; Phenomenex, Aschaffenburg, Germany). After passing the column, residual metabolites were eluted with 180 µL MeOH/H_2_O (80/20, v/v). The combined eluates were taken to dryness in a centrifugal evaporator. Samples were reconstituted in 100 µL of 5 mM NH_4_OAc in CH_3_CN/H_2_O (50/50, v/v). LC/MS analysis was performed on a Thermo Scientific Dionex Ultimate 3000 UHPLC system connected to a Q Exactive mass spectrometer (QE-MS) equipped with a HESI probe (Thermo Scientific, Bremen, Germany).

Chromatographic separation was achieved by applying 3 µL sample on a SeQuant ZIC-cHILIC column (100 × 2.1 mm, 3.5 μm) (Merck, Darmstadt, Germany), protected by a Supelco ColumnSaver particle filter (Merck) and a gradient of mobile phase A (5 mM NH_4_OAc in CH_3_CN/H_2_O [40/60, v/v]) and mobile phase B (5 mM NH_4_OAc in CH_3_CN/H_2_O [95/5, v/v]), maintaining a flow rate of 200 µL min^−1^ and a column temperature of 45 °C. The LC gradient programme was 100% mobile phase B for 2 min, followed by a linear decrease to 20% B within 23 min, maintaining 20% B for 21 min and returning to 100% B in 2 min, followed by 7 min 100% B for column equilibration before each injection. The eluent was directed to the QE-MS from 2.7 min to 46 min after sample application.

Mass detection was conducted in alternating pos./neg. full scan mode (at 70 k resolution, scan range m/z 69–1000, AGC target 1E6 and 200 ms max. injection time). HESI parameters: Sheath gas: 20, aux gas: 1, spray voltage: 3.0 kV, capillary temperature: 300 °C, S-lens RF level: 50.0, aux gas heater temperature: 120 °C. Manual curation and integration of chromatographic peaks were performed with TraceFinder 3.3 using a mass tolerance of ±2 mMUs. Labeling was calculated after correction for natural isotopologue distribution using the FluxFix application (http://fluxfix.science).

### Seahorse metabolic flux analysis

The effect of RBC ghosts on HCMEC extracellular acidification rate (ECAR) and oxygen consumption rate (OCR) was examined using the Seahorse XF96e Extracellular Flux analyzer (Agilent Technologies), following the manufacturer’s protocols. Briefly, HCMECs were plated onto XF96 (V3) polystyrene cell culture plates (Agilent Technologies) in MV2 medium. After 24 h of incubation under standard growth conditions, the growth medium was exchanged with the XF assay medium (XF RPMI with 1 mM HEPES [Agilent Technologies] supplemented with 1 mM pyruvate, 2 mM L-glutamine, and 10 mM glucose), and cells were incubated at 37 °C in a SpectraMax i3 (VWR; INCU-Line) non-CO_2_ incubator for 45 min prior to performing the assay. After calibration and initialization of the sensor cartridge, three baseline measurements and three response measurements after the addition of each compound were taken. For the Real-Time ATP Rate Assay, sequential injections of ATP Synthase inhibitor oligomycin (final concentration 1.5 µM), the complex 1 inhibitor rotenone and the complex 3 inhibitor antimycin A (0.5 µM each) were applied. For Mito Stress Tests, oligomycin (1.5 µM), FCCP (1 µM) and rotenone/antimycin A (0.5 µM each) were used. ECAR and OCR are reported as absolute rates (mpH min^−1^ for ECAR and pmol min^−1^ for OCR), normalized against cell counts (determined using 20 µM Hoechst 33,342; Thermo Scientific, 62,249). Detection was performed with the Cytation 5 (BioTek) multimode reader using image-based autofocus in the channel of the DAPI filter cube (377/360 nm excitation and 447/460 nm emission). Data analysis was performed using Wave 2.6.3.5 Software (Agilent Technologies).

### Angiogenesis assays

HCMECs were cultured on 0.2% gelatin-coated tissue culture in endothelial cell basal medium MV2 (PromoCell, C-22221) with supplement mix (PromoCell, C-39221). At 80–90% confluency, cells were harvested using 0.05% trypsin–EDTA (Gibco, 25,200,056), counted using hemocytometer and the cell number adjusted for each experiment, as specified below. For the Matrigel angiogenesis assay, 10^4^ HCMECs were plated in 24-well plates coated with 350 µL of Matrigel (Corning, 356,237) and cultivated in serum-reduced medium (Opti-MEM™; Gibco, 31,985–047), alone or in the presence of lysed RBCs fractions. Following incubation for five hours, the angiogenic response was recorded by capturing images on a computer-assisted inverted microscope (Motic, AE31 series). The total length of the endothelial tubule network and the number of branch points (nodes) were measured using Olympus cellSens [Ver.2.3] imaging software. Endothelial spheroids were generated using the hanging drop method with a density of 10^4^ cells mL^−1^ in MV2 medium containing 20% methylcellulose (8% stock prepared in 10X M199 medium; Gibco, 11,825,015). Cells were incubated overnight on an inverted square petri dish (Greiner BIO-ONE) allowing for spheroid formation. The next day, type I rat tail collagen (Corning, 354,236) was prepared by diluting it (at a 1:1 ratio) with 0.1% acetic acid solution, mixed with 10X M199 medium, and neutralized just before use with 0.2 N NaOH. The spheroids were harvested and suspended in a methylcellulose solution supplemented with 5% FCS and gently mixed in a 1:1 ratio with the collagen working solution. The spheroid suspensions were evenly distributed into 24-well plates (1 mL per well) and incubated at 37 °C for 30 min to allow the collagen to polymerize. After solidification, MV2 medium, alone or containing RBC ghosts as well as inhibitors or stimulators, was added to the wells and incubated for 16 h at 37 °C. Microscopic images of at least 5–10 spheroids per variable were randomly captured. The total sprout length and the number of sprouts per spheroid were determined in a blinded manner using Olympus cellSens [Ver.2.3] imaging software.

### Scratch wound closure assay

An equal number of HCMECs was seeded onto 6-well culture dishes and grown until 95–100% confluency. To examine endothelial migration, a scratch wound was induced using a sterile 100 μL pipette tip. Cell debris was removed by a brief wash with PBS. Then, MV2 medium as well as RBC ghosts were added, and cells were incubated in a tissue culture incubator. Images were acquired immediately following the scratch and after five hours of incubation (at 37 °C, 5% CO_2_). Cell migration over time was assessed by manually counting the number of cells migrated into the scratch wound (at 20X magnification) and by automatically measuring the cell-free area (and expressing it as % of the area at baseline, set as 100%) using Image J software.

### Immunofluorescence microscopy

HCMECs were cultured on 0.2% gelatin-coated glass coverslips or chamber slides and fixed with freshly prepared paraformaldehyde (PFA; 4% in 1X PBS, pH 7.5) for 15 min. Fixed cells were washed with 1X PBS containing 0.1 M glycine to block unreacted aldehydes followed by permeabilization with 0.1% Triton X-100 for 5 min. Cells were immunostained with antibodies against COX2 (Abcam, ab179800, RRID:AB_2894871), Ki-67 (Abcam, ab15580, RRID:AB_443209), NFκB p65 (Cell Signaling Technology, 6956, RRID:AB_10828935), VE-cadherin (Santa Cruz Biotechnology, sc-9989, RRID:AB_2077957) or VEGF (Abcam, ab52917, RRID:AB_883427) for 60 min at RT, followed by goat anti-rabbit IgG, Alexa Fluor™ Plus 555 secondary antibodies (Thermo Fisher Scientific, A32732). Fluorescein phalloidin (Thermo Fisher Scientific, F432) was used to stain F-actin fibers, 4',6-diamidine−2'-phenylindole dihydrochloride (DAPI; Sigma-Aldrich, D9542) to stain nucleic acids. Cells were imaged on an inverted fluorescence microscope (Keyence, BZ-X810) using BZ-X800 image software.

### TNP-ATP loading

Membrane-associated ATP-binding sites were detected by incubating RBC ghosts with 1.5 mM fluorescent nucleotide analog 2',3'-O-Trinitrophenyl-adenosine−5'-triphosphate, Triethylammonium salt (TNP–ATP; Jena Bioscience, Cat. NU-221) for 30 min at 37 °C in buffer (10 mM Tris, pH 7.5, 40 mM NaCl, 2 mM MgCl_2_ and 0.25 mM EDTA. The RBC ghosts were washed once with buffer containing 17 mM Tris (pH 7.5). Binding-induced fluorescence was observed on a confocal microscope (Leica TCS SP8) under a 60X oil immersion objective lens and evaluated using Leica Application Suite X software (LAS X).

### Intracellular ATP detection

ATP levels in RBC ghosts were measured using the ATP-Red cell-permeable ATP sensor (BioLegend, Cat. No. 421944) according to the manufacturer’s instructions. In short, RBCs ghosts were isolated from purified intact RBCs, washed once with ice-cold phosphate-buffered saline (PBS) and incubated with ATP-Red dye for 10 min at 37 °C. Intracellular ATP levels were quantified by flow cytometry following staining. Fluorescence was detected in the PE channel. Intracellular ATP levels were expressed as mean fluorescence intensity (MFI).

### Cytochemical staining of iron

Iron or iron metabolites were visualized by incubating 4%PFA-fixed ECs or murine spleen tissue for 30 min in a 1:1 (vol:vol) mixture of 10% potassium hexacyanoferrate (II) (Sigma, P-3289) and 20% HCl (Applichem, A3397,1000) followed by counterstaining with nuclear fast red (Roth; N069-1). Cells were fixed using 4% PFA for 15 min, and photographed using an inverted microscope (Motic AE31) and Motic image software.

### Single-cell Foerster resonance energy transfer (FRET) imaging

Single-cell FRET analysis was essentially performed as previously described, however, the protocols were adapted to HCMVECs [51]. In brief, HCMVECs were plated on 0.2% gelatin-coated 24 mm glass coverslips (Roth, KHX8.1). At 80% confluency, cells were transfected with the plasmid encoding the cAMP biosensor (Epac-S^H187^) using Lipofectamine™ 2000 Transfection Reagent (Invitrogen, 11,668,019) according to the manufacturer´s instructions. After 48 h of transfection, coverslips with transfected cells were loaded onto imaging chambers (Attofluor™, ThermoFisher Scientifics) and washed with imaging buffer (144 mM NaCl, 5.4 mM KCl, 2 mM CaCl_2_ (all from Carl Roth), 1 mM MgCl_2_ (AppliChem), 10 mM HEPES (Sigma-Aldrich); pH 7.3) twice before imaging, and then mounted on the microscope. Experiments were performed at room temperature on an Olympus IX83 inverted microscope (Olympus, Hamburg, Germany) equipped with an oil immersion objective (UPLAPO60XOHR Objective 60x/1,5), a H 488 LPXR superflat Vers.2 beam splitter (AHF analysentechnik, Tübingen, Germany), a Lumencor New Spectra X LED lightsource (Lumencor, Beaverton, OR, US), and an ORCA-FUSION CMOS-Camera (C14440-20UP, Hamamatsu Photonics, Shizuoka Pref., Japan) with an Optosplit II dual emission image splitter (Cairn research, Faversham, UK). The system was controlled using Micromanager Software. The fluorescent donor was excited at 438 nm for 50 ms every 10 s and fluorescent images in the donor and acceptor emission channels (475 nm and 542 nm, respectively) were recorded every 10 s. Raw emission intensities were background-corrected by subtracting the fluorescence intensity of a cell-free region. Further, bleed-through of donor emission into the acceptor channel was subtracted as described previously (Börner et al., 2011).

After reaching a stable baseline, cells were stimulated with RBC ghosts or ATP (100 µM), followed by forskolin (10 µM)/IBMX (100 µM) (Cayman Chemical, 11,018; Sigma, I5879 respectively) at the end of every experiment to record maximal cAMP levels. Inverted FRET ratios (mTq2/FRET) were calculated and normalized to baseline (average of 10 data points before RBC or ATP addition set to 0% and forskolin/IBMX set to 100%). After every experiment, an image of direct cpVenus excitation at 505 nm (emission: 560 nm) was captured to evaluate the expression levels of the sensors and to calculate bleedthrough.

Pseudocolored FRET ratio images were obtained by background subtraction, masking, and correction of acceptor-channel spectral bleed-through prior to perform intensities ratio using the emission recorded at different timepoints (800 s for baseline; 3500 s for ATP stimulation; 4600 s for FSK/IBMX maximal stimulation).

### cAMP assay

To measure cAMP levels in HCMVECs, cells were cultured on the 0.2% gelatin-coated tissue culture grade plates (Greiner Bio-One, 664,160) in MV2 medium with supplement mix (PromoCell). Cyclic AMP was measured using the HTRF cAMP Gs Dynamic Kit (Revvity, 62AM4PEB), following the protocol provided by the manufacturer. At 80–90% confluency, cells were treated with and without 50 µg mL^−1^ RBC ghosts for two hours, or with ATP (100 µM; Sigma, A6419) To prevent cAMP hydrolysis during the treatment, culture medium (MV2) was supplemented with IBMX (100 µM) (Sigma, I5879). Forskolin (10 µM) was used as a positive control for maximal cAMP production. By the end of the treatment, cells were collected using cell scraper in the cold 1X PBS supplemented with 100 µM IBMX and pelleted at 300 g for five min at 4 °C. The cell pellet was resuspended in 1X stimulation buffer, and the reaction was terminated by adding 5 μL lysis buffer containing cAMP-d2 and 5 μL lysis buffer containing Anti-cAMP-cryptate, per well. cAMP levels were measured using a PHERAstar fsx plate reader (BMG Labtech, Ortenberg, Germany), equipped with a HTRF Filter cube (BMG Labtech, 620/665 nm emission). Results were normalized to the total protein content of each sample.

### Statistical analysis

Statistical analyses were performed using data analysis software (GraphPad Software, Inc.; version 10.0.0). Data are presented as means with errors bars indicating the standard error of the mean (SEM) or as median and interquartile range, depending on the presence of normal distribution, as tested using the Shapiro–Wilk test. For the comparison of two groups, two-tailed unpaired Student’s t-test (Figs. 3E, G, H, J, L; 4A; S3A, B; S5C) or Mann–Whitney *U* test (Fig. 4J) were used, depending on the presence of normal distribution or not. If more than two groups were compared, one-way ANOVA followed by Sidak's multiple comparisons test (Figs. 2C, D, G, I, J; 3C, N; 4B, H; 5D, H, J, N; 6F, J; 7G; S2B, C, F; S6B, D; S7B, D; S8B–D and S9A, C) or Kruskall-Wallis, Dunn's multiple comparisons test (Figs. 4L; 5I, K; 6D; S2E; S6F; S9B) were employed. Differences between two groups and an additional variable (e.g. time) in Figs. 4B, S5A, B were determined using uncorrected two-way ANOVA, mixed-effects model, Sidak’s multiple comparisons test. Statistical differences were assumed if P reached a value less than 0.05. Non-significant differences are not shown.

## Supplementary Information

Below is the link to the electronic supplementary material.

Supplementary file1 (PDF 1974 KB)

Supplementary file2 (DOCX 15 KB)

## Data Availability

All unique/stable materials and protocols generated in this study are available from corresponding author without restriction, but requiring reasonable compensation by requestor for its processing and shipping or a completed Materials Transfer Agreement if there is potential for commercial application. All of the data that support the findings of this study are included in the article. Any additional information required to reanalyze the data reported in this paper is available from the corresponding author contact upon request.

## Abbreviations

AC: Adenylyl cyclase
AP-1: Activator protein-1
ATP: Adenosine triphosphate
ChIP-seq: Chromatin immunoprecipitation sequencing
DEG: Differentially expressed gene
DZHK: Deutsches Zentrum für Herz-Kreislauf-Forschung
EC: Endothelial cell
FDR: False discovery rate
FRET: Fluoresence resonance energy transfer
DO: Disease ontology
GO: Gene ontology
GPCR: G-protein coupled receptor
HCMEC: Human cardiac microvascular endothelial cell
PAD: Peripheral artery disease
PDE: Phosphodiesterase
RBC: Red blood cell
