# Supplementary material for: Lysed erythrocyte membranes metabolically prime endothelial cells for angiogenesis
Source: Angiogenesis. 2026 Sep 25;29(4):74. doi: 10.1007/s10456-026-10096-y (PMC13615041; doi:10.1007/s10456-026-10096-y)
Supplement: Supplementary file 1 [file 10456_2026_10096_MOESM1_ESM.pdf]

Figure S1

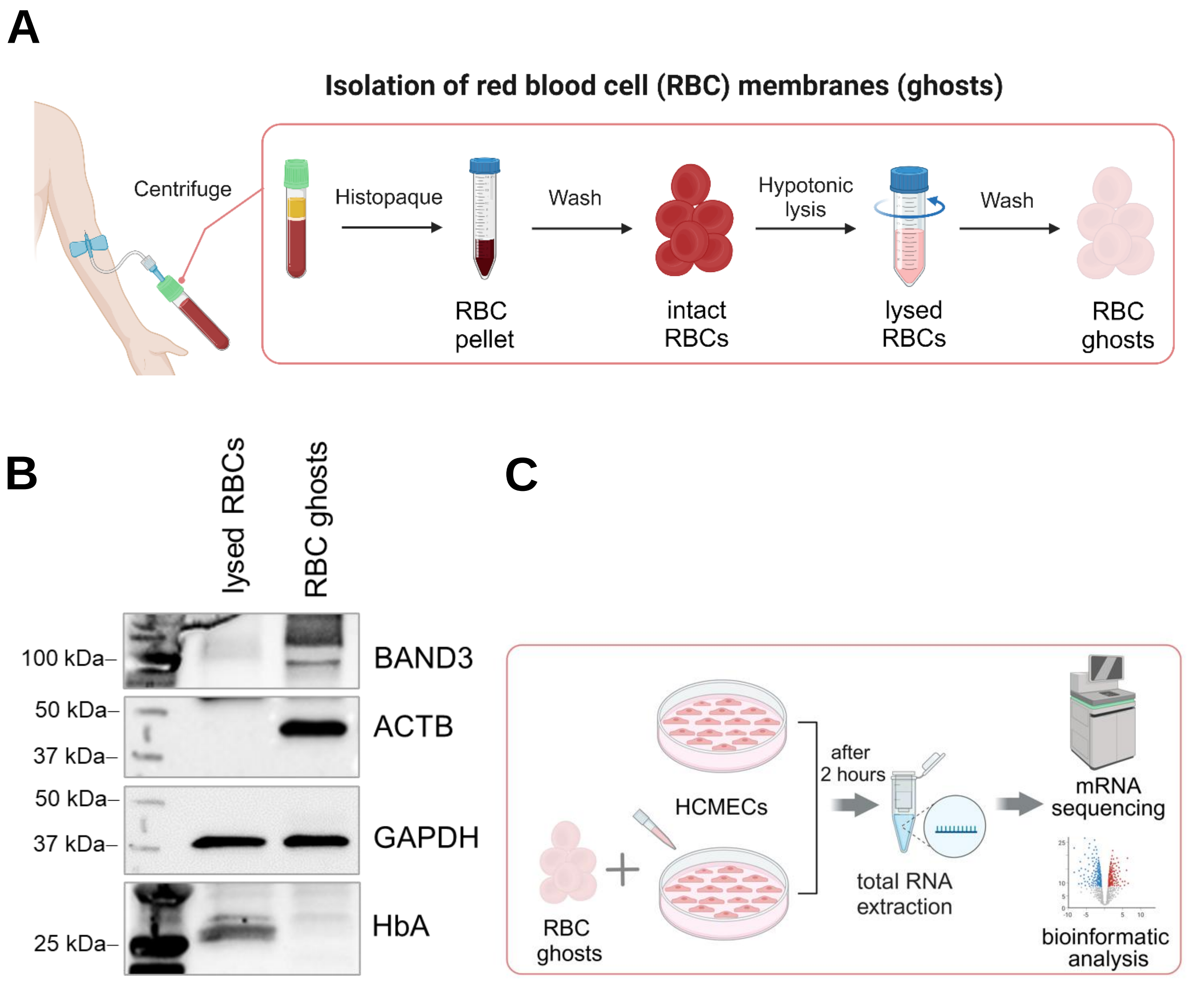

Figure S1. Experimental work-flow and isolation of hemoglobin-free human erythrocyte membranes.

A Schematic drawing illustrating the isolation of lysed red blood cell (RBC) membranes, so called RBC ghosts, from human peripheral venous blood. This panel was generated by BioRender.com.

B Immunoblot membrane showing the presence of hemoglobin (HbA) in lysed RBCs and its absence in their membrane fraction. Equal amounts of protein were loaded for comparison of relative expression levels. Beta-actin and the anion exchanger (AE1, also known as SLC4A1 or BAND3) are expressed in the RBC membrane, GAPDH is expressed in the RBC membrane and the RBC cytosol.

C Schematic drawing showing the experimental work-flow of preparing human cardiac microvascular ECs, control or RBC ghost treated for two hours, for bulk RNA-sequencing. This panel was generated by BioRender.com.

Figure S2

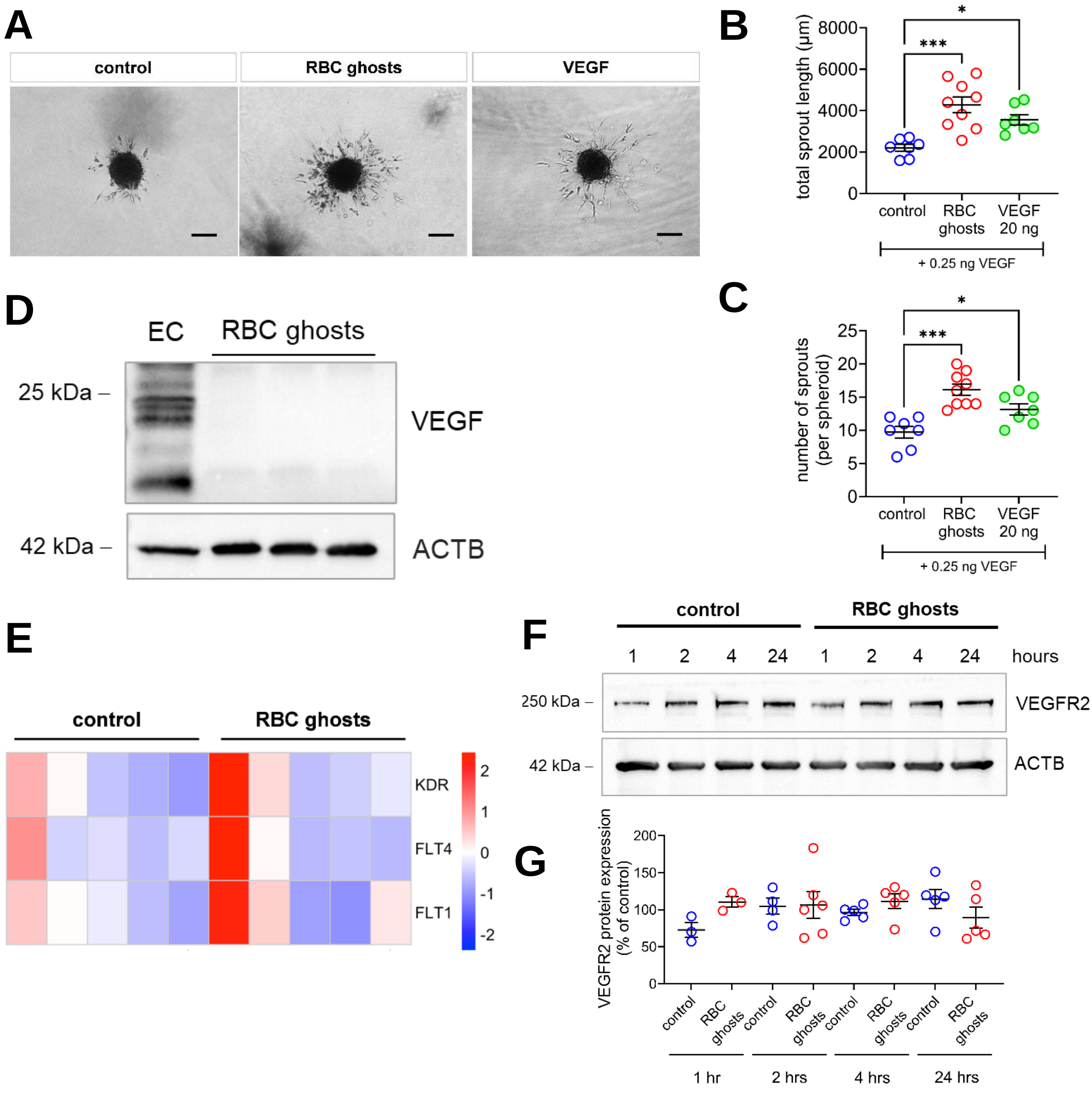

Figure S2. Source of VEGF and endothelial VEGFR expression

A-C Spheroid angiogenesis assay. Representative images (**A**) and quantification of the number of sprouts per spheroid (**B**) and the total network length (**C**). Mean findings from n=2 RBC donors per condition, with multiple spheroids analyzed per condition for each donor. Each dot represents one individual spheroid. VEGF (20 ng) was used as positive control. Scale bars, 200 µm. *P<0.05 and ***P<(one-way ANOVA, Sidak's multiple comparison test).

D, Immunoblot membrane showing VEGF protein expression in human endothelial cells (EC) and its absence in human red blood cell membranes (RBC ghosts). Beta-actin (ACTB) was used as a protein loading controls.

E, Heat map showing RNA-seq expression of VEGFR1 (FLT1), VEGFR2 (KDR) and VEGFR3 (FLT4) in HCMECs, untreated (control) or incubated with RBC ghosts for 2 hours. (n=5 biological replicates per group) Colors represent row-scaled normalized expression (Z-scores), with red and blue indicating relatively higher and lower expression, respectively.

F, G VEGFR2 protein expression in HMCECs, untreated (control) or RBC ghost-treated for 1, 2, 4 and 24 hours. Representative membrane (**F**). Quantitative analysis (**G**). (one-Way ANOVA, Sidak's multiple comparisons test). (n=3-6 biological replicates per group)

Figure S3

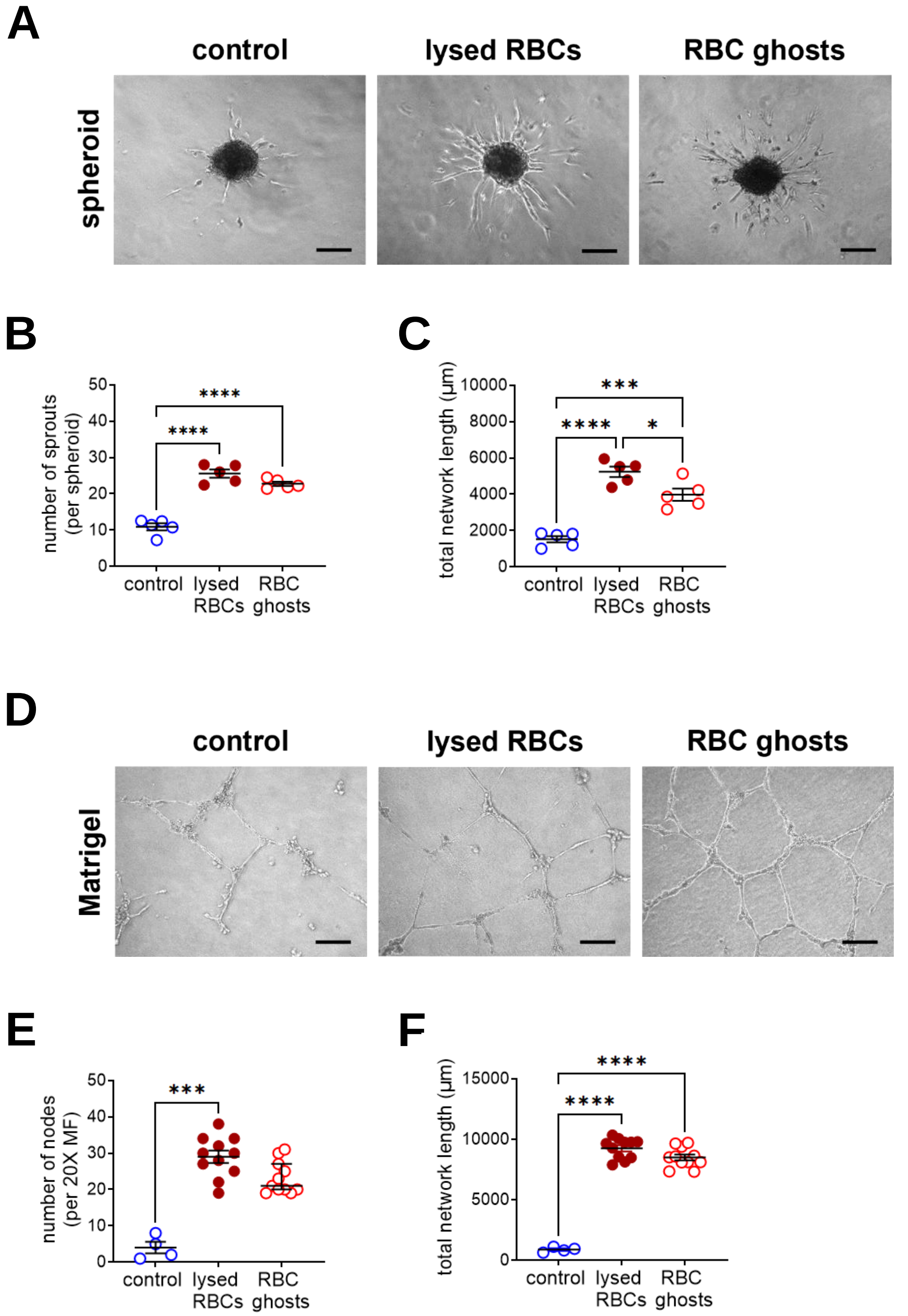

Figure S3. The proangiogenic mediators are localized in the erythrocyte membrane fraction. ECs were incubated overnight with equal amounts of lysed RBCs or only their membrane fraction (RBC ghosts) and compared to untreated cells (control).

A-C Spheroid angiogenesis assay. Representative images (**A**) and quantification of the number of sprouts per spheroid (**B**) and the total network length (**C**). Mean findings in more than five spheroids from n=5 RBC donors per condition. Scale bars, 200 µm.

D-F Matrigel™ angiogenesis assay. Representative images (**D**) and quantification of the number of nodes per 20X microscope field (MF; **E**) and the total network length (**F**). n=3 RBC donors with at least three or more images collected per sample, per condition. Scale bars, 200 µm.

*P<0.05, ***P<0.001 and ****P<0.0001. P values were calculated using one-way ANOVA, Sidak's multiple comparison test (B, C, F) or Kruskall-Wallis, Dunn's multiple comparison test (E).

Figure S4

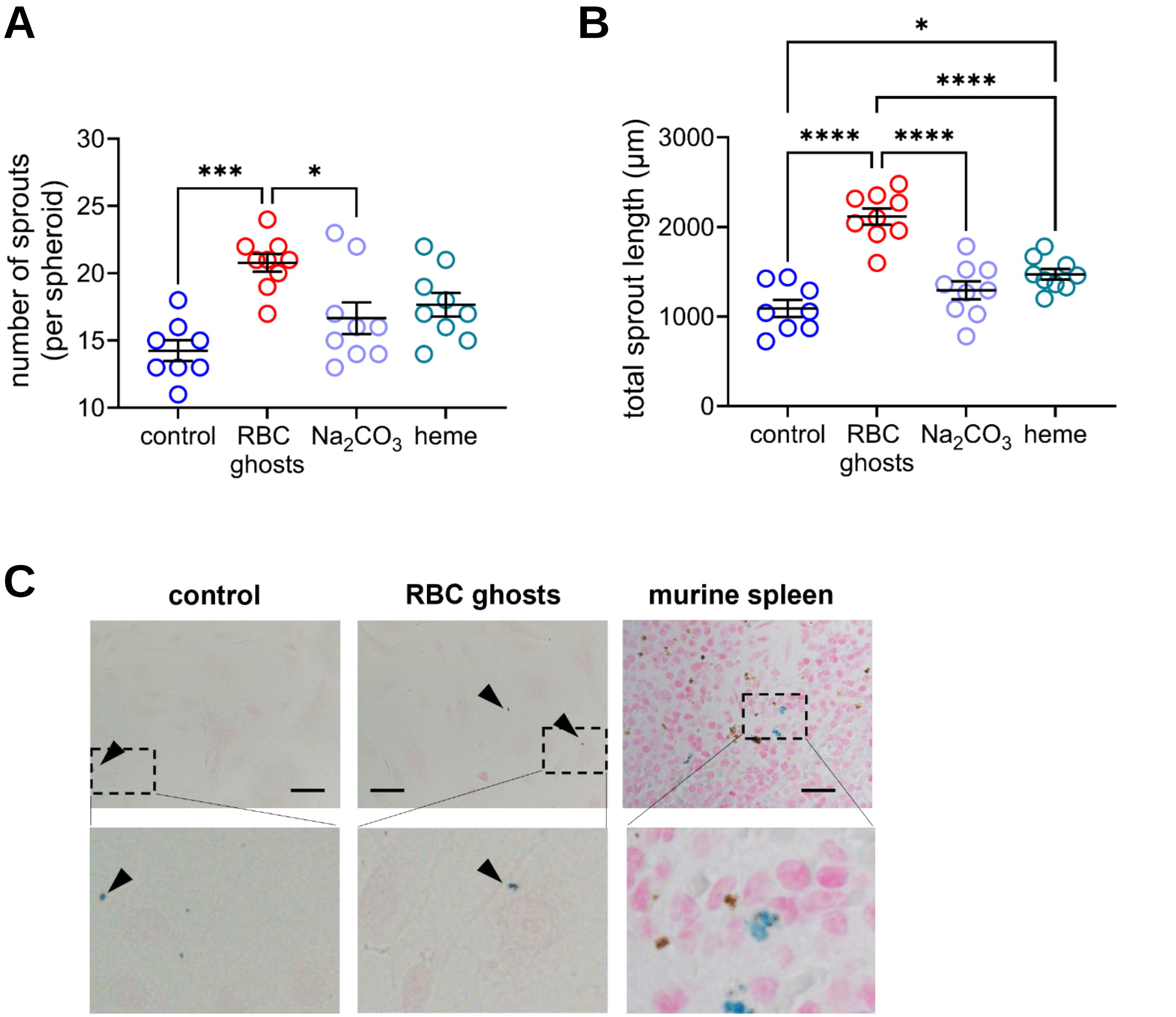

Figure S4. Heme isolated from lysed erythrocytes does not induce endothelial sprout formation.

Heme was isolated from whole blood, as described in the Methods, and dissolved in 0.1 M Na_2CO_3 (at a concentration of 50 µM mL^{-1}). MV2 medium supplemented with an equal volume of Na_2CO_3 alone was used as control to heme. Control spheroids were cultured in MV2 medium.

A, Number of sprouts per spheroid. **B,** Total sprout length. (n=3 biologically replicates (RBC donors) with multiple spheroids analyzed per donor, per condition. Each dot represents one individual spheroid) *P<0.05, ***P<0.001 and ****P<0.0001 (one-way ANOVA, Sidak's multiple comparisons test.

C, Representative images of HCMECs incubated with RBC ghosts for 24 hours and stained with Perl's iron to detect iron (blue signal; arrowheads) or its metabolite hemosiderin (yellow-brown signal). Findings in murine spleen are shown as positive control. Images in the bottom row show details from areas selected from images in the top row (indicated by black boxes). Scale bars, 100 µm.

Figure S5

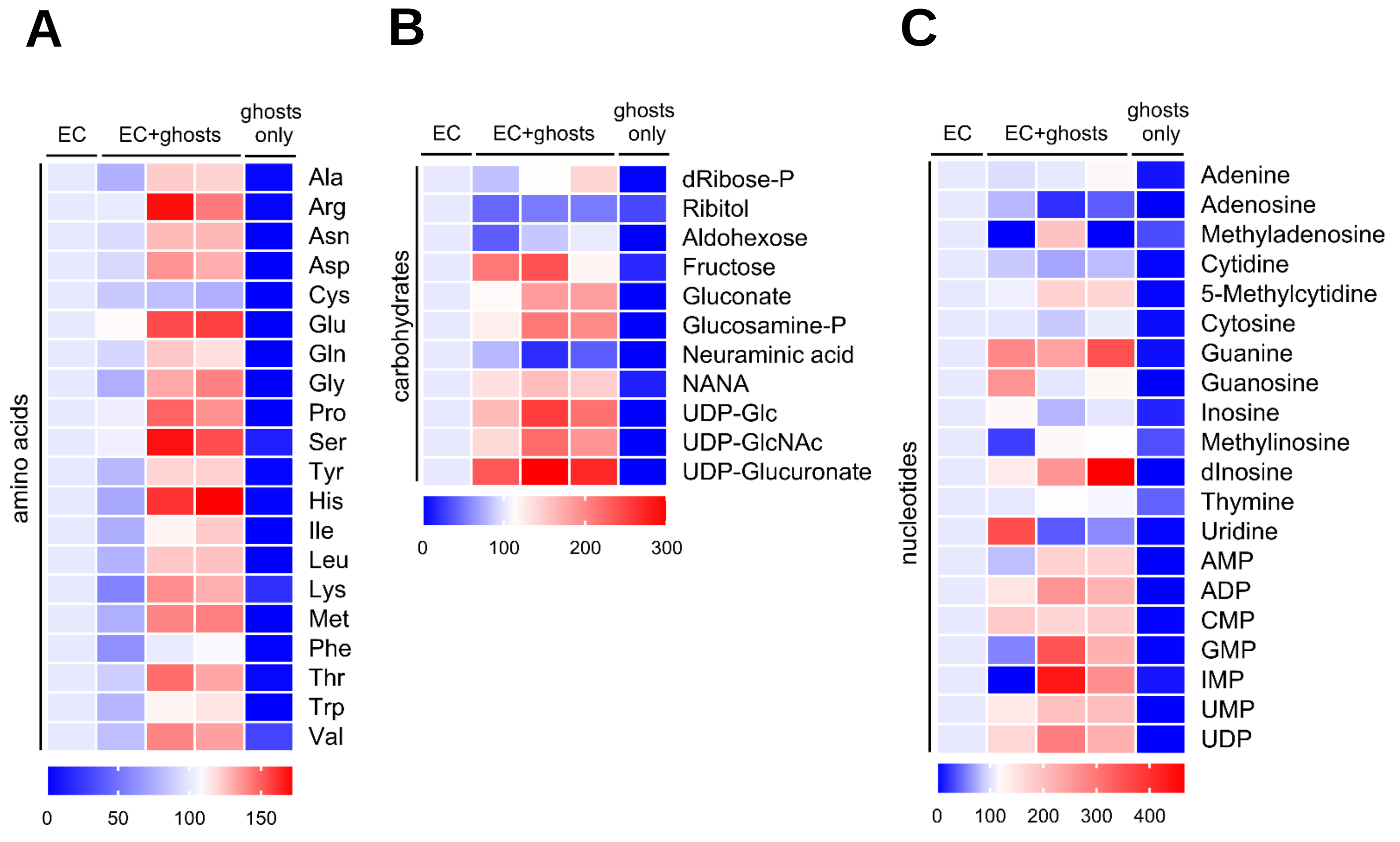

Figure S5. Lysed erythrocyte membranes promote the generation of biosynthetic precursors.
Heat-maps showing the results of metabolomic analysis using LS/MS. Determination of metabolite levels of amino acids (**A**), carbohydrates (**B**), and nucleotides (**C**) in the water-soluble fraction of cellular extracts from human ECs, alone or after incubation with human RBC ghosts (n=3 biologically independent samples) for two hours. RBC ghosts alone (without ECs) were also examined. Data (rel. norm. peak area) are expressed as the percentage of untreated control ECs (set at 100%; white color). Lower values are blue, higher values are red.

Figure S6

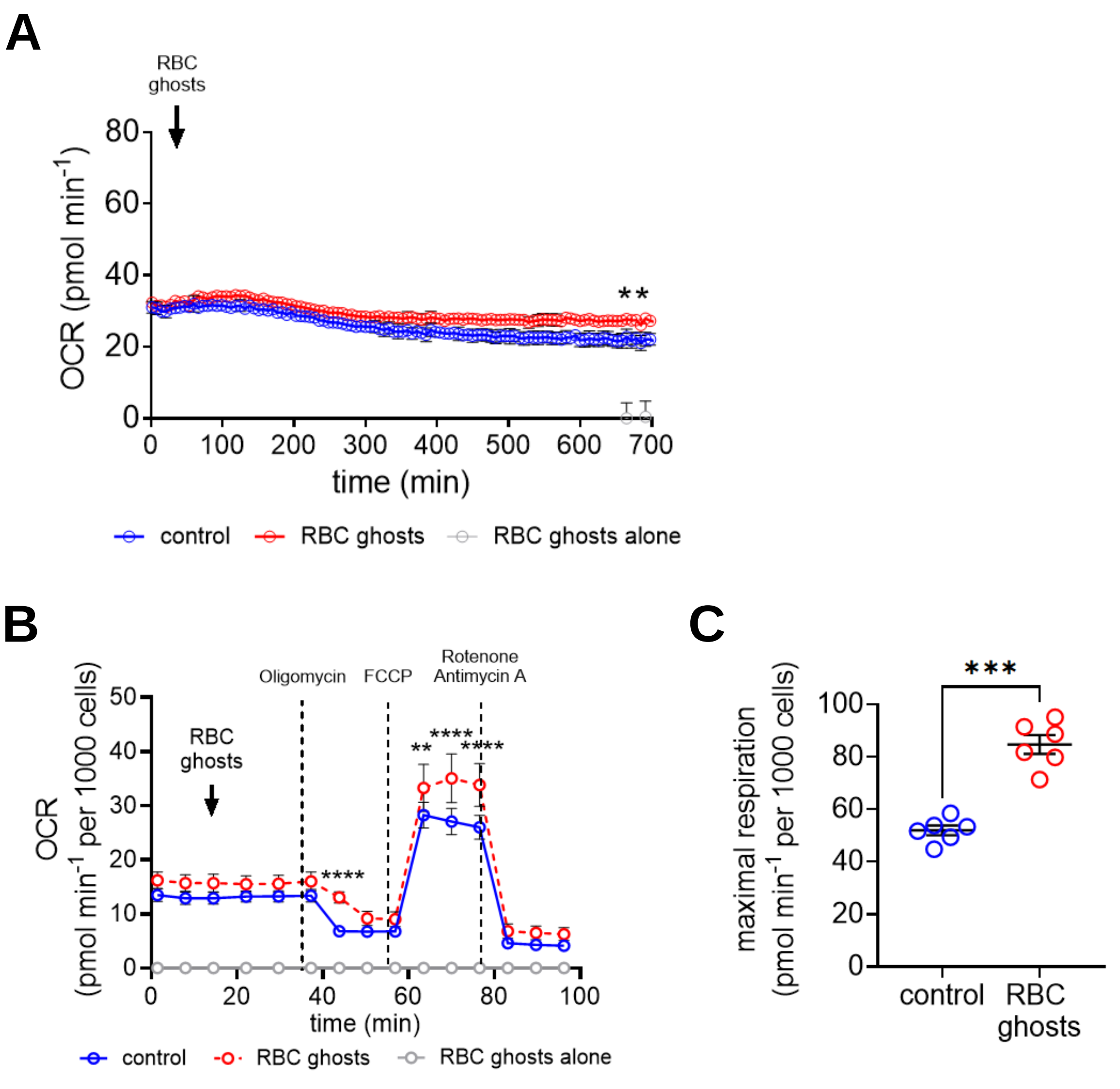

Figure S6. Effects of lysed erythrocyte membranes on endothelial oxidative phosphorylation

Oxygen consumption rate (OCR) in ECs, control-treated or exposed to lysed erythrocyte membranes (RBC ghosts). RBC ghosts alone without ECs were also examined. Real-time measurements, at baseline (**A**) and during the oxidative stress test (**B**). n=6 biologically independent samples. The time point of RBC addition is indicated with an arrow. **P<0.01 and ****P<0.0001 (two-way ANOVA, Sidak's multiple comparisons test; n=4-5). FCCP, carbonyl cyanide-4 (trifluoromethoxy)phenylhydrazone. **C,** Summary of the maximal respiration. ***P<0.001 (Student's t test).

Figure S7

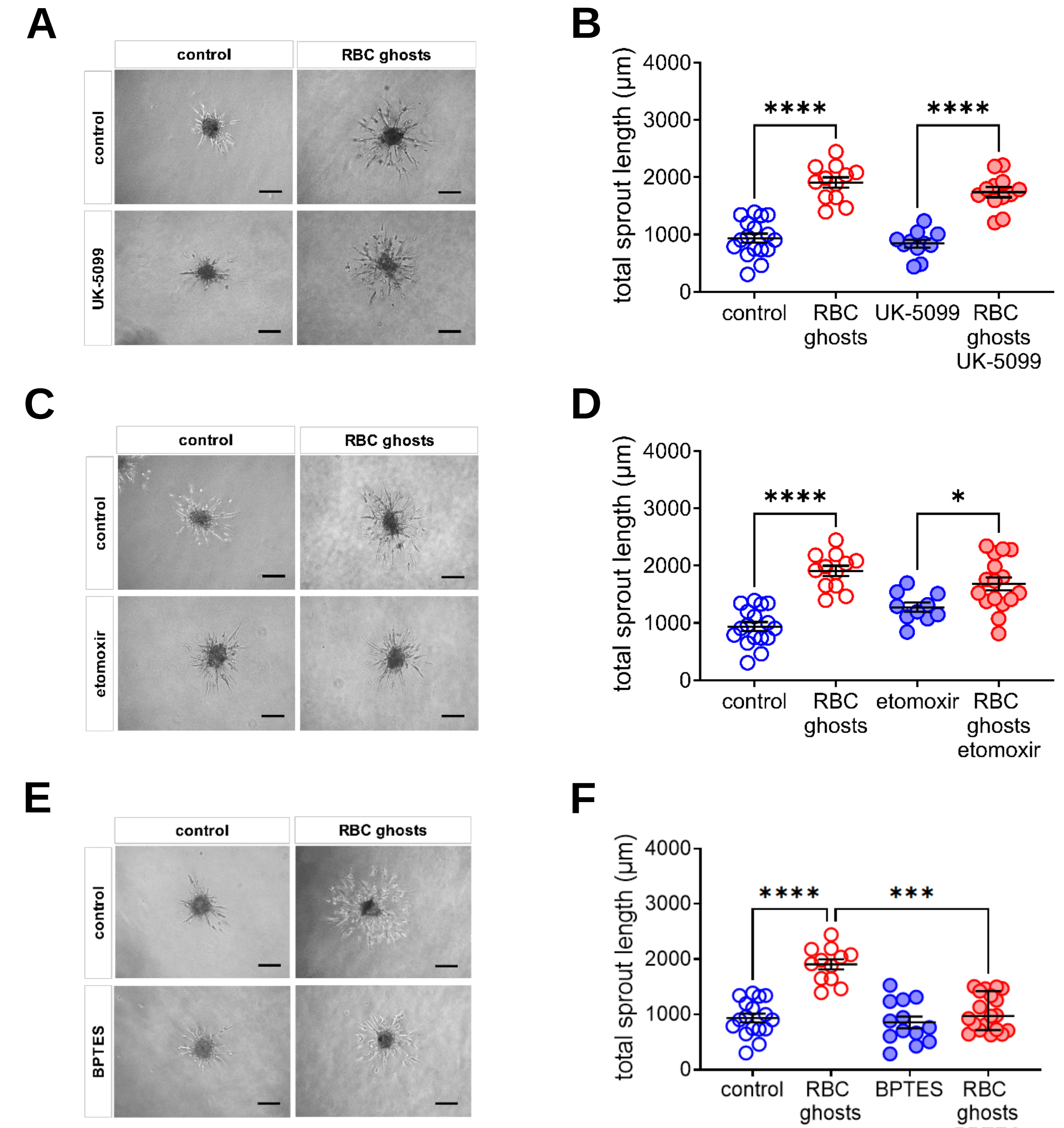

Figure S7. Inhibition of glutaminolysis, but not of mitochondrial pyruvate transfer or fatty acid oxidation prevents the effects of lysed erythrocyte membranes on endothelial sprout formation.

Spheroid angiogenesis assay to examine the effects of RBC ghosts on sprouting angiogenesis.

A, B UK-5099 (20 µM) was used to inhibit mitochondrial pyruvate transfer;

C, D Etomoxir (100 µM) was used to inhibit fatty acid oxidation;

E, F BPTES (15 µM) was used to inhibit glutaminolysis.

Representative images and quantitative analysis of total sprout length (n=3 biologically independent RBC samples with multiple spheroids analyzed per sample, per condition for each donor. Each dot represents one individual spheroid). Scale bars, 200 µm. Note that UK-5099 (B), etomoxir (D) and BPTES (F) were examined in the same experiment and share the same 'control' and 'RBC ghosts' group. P values were calculated using one-way ANOVA, Sidak's multiple comparisons test (B, D) or Kruskal-Wallis test, Dunn's multiple comparisons test (F). *P<0.05, ***P<0.001 and ****P<0.0001.

Figure S8

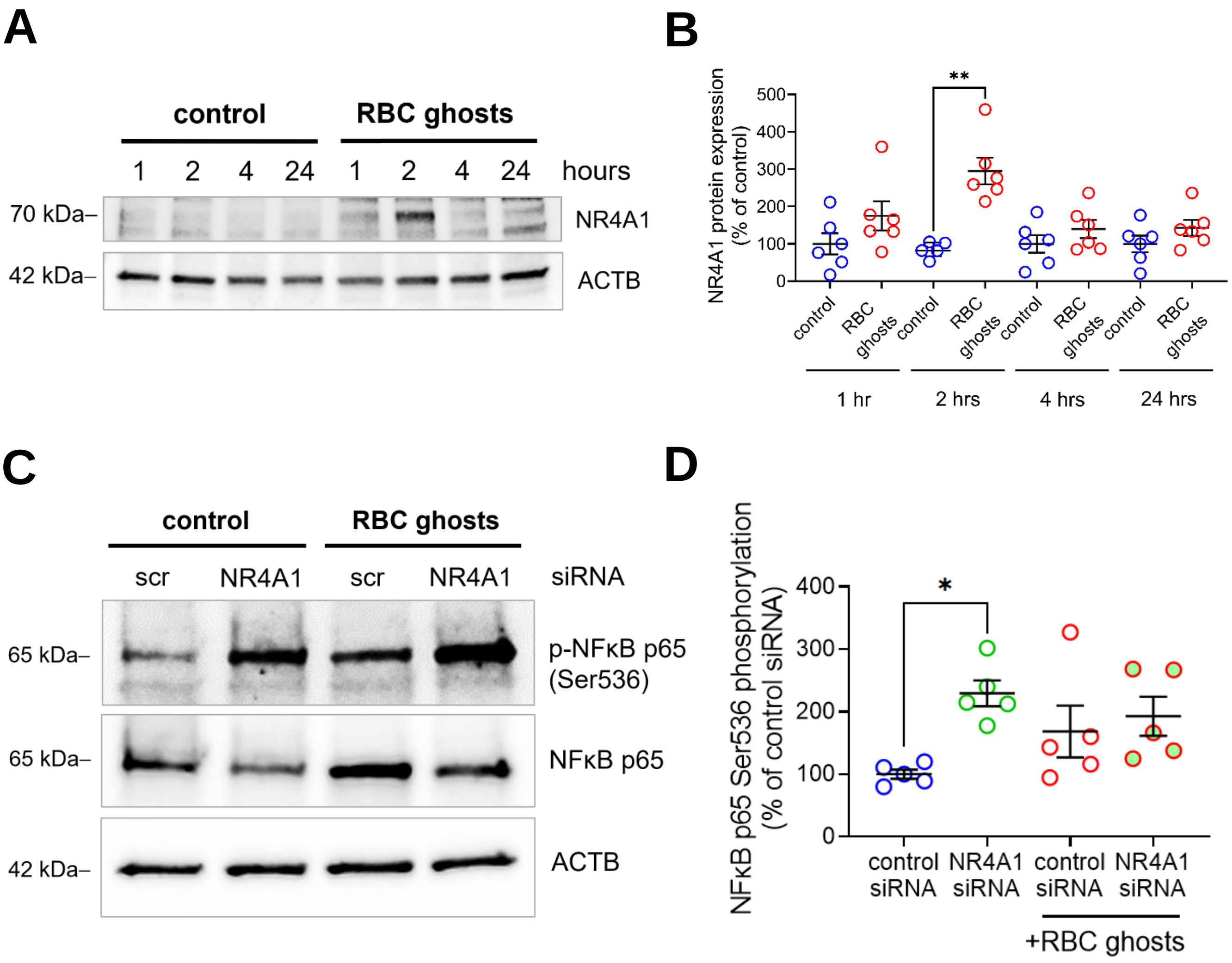

Figure S8. Endothelial NR4A1 expression following exposure to lysed erythrocyte membranes and effects of siRNA-mediated NR4A1 downregulation on NFκB activation

A, B Time course analysis of NR4A1 protein expression in ECs, control-treated or incubated with RBC ghosts for the indicated time points. Representative immunoblot membrane (A). Quantitative analysis of n=5-6 independent biological replicates (RBC donors) (B). **P<0.001 (one-way ANOVA, Sidak‘s multiple comparisons test).

C, D siRNA-mediated downregulation of NR4A1 in ECs and its effect on NFκB 65 subunit phosphorylation at Ser356. Representative immunoblot membrane (C). Quantitative analysis of n=5 independent experiments in (D). *P<0.05 (one-way ANOVA, Sidak‘s multiple comparisons test).

Figure S9

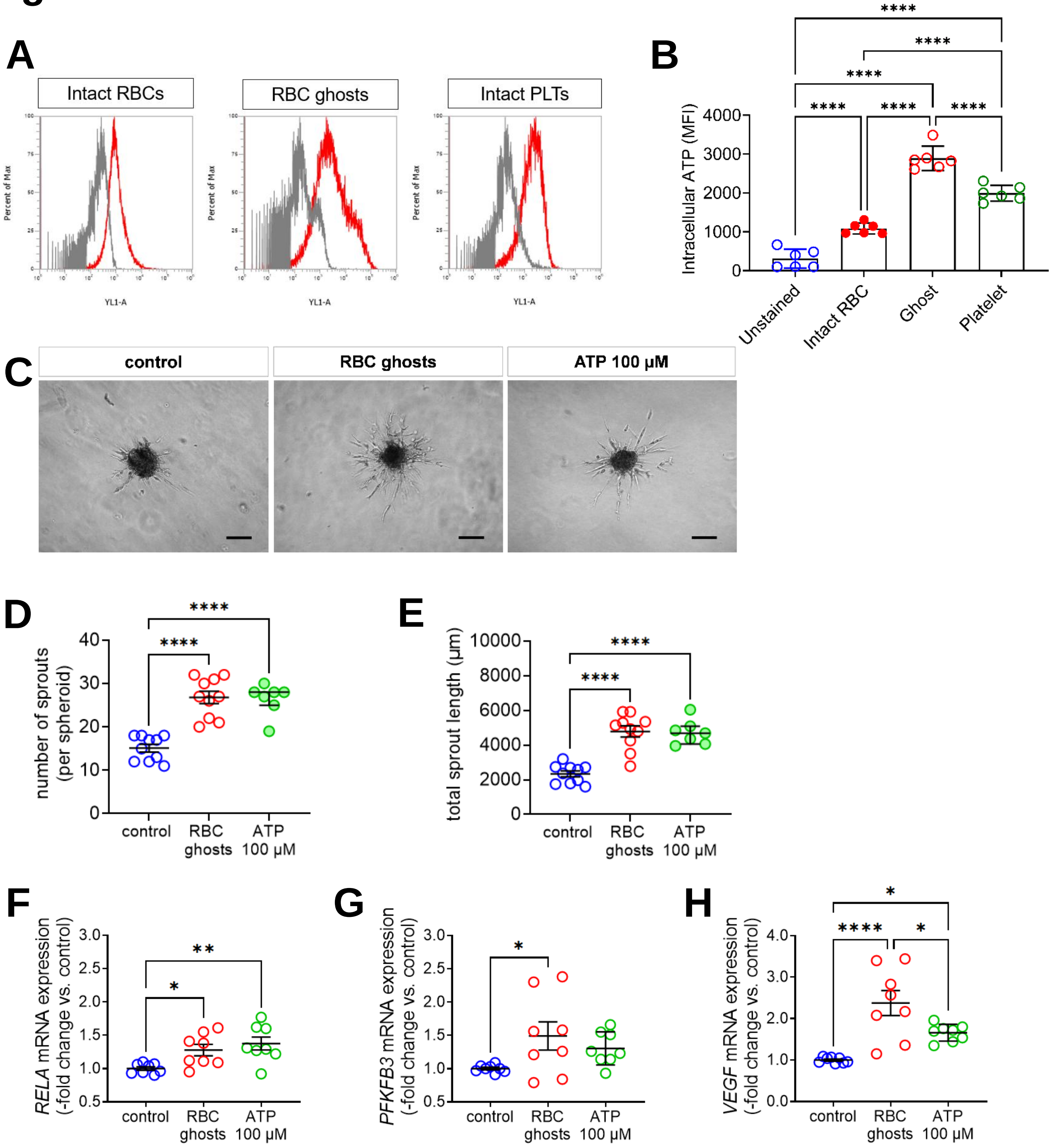

Figure S9. RBC membrane ATP as causal agent.

A, B Representative histogram (A) and quantitative analysis (B) after flow cytometry quantification of intracellular ATP using BioLegend ATP-Assay-Kit. Unstained cells are used as negative control, platelets (PLTs) as positive control. ****P<0.0001 (one-way ANOVA, Sidak's multiple comparisons test), n=5 biologically independent samples.

C-E Spheroid angiogenesis assay. Representative brightfield images (C) and quantification of the effects of RBC ghosts and ATP (100 µM) on endothelial sprout formation (D) and tube elongation (E). Scale bars, 200 µm. ****P<0.0001 (one-way ANOVA, Sidak's multiple comparisons test in D & E). (n=2 biologically independent RBC samples with five to ten images analyzed per sample, per condition) **F-H** qPCR analysis of *RELA* (F), *PFKFB3* (G) and *VEGF* (H) mRNA expression in untreated ECs (control) or ECs treated with RBC ghosts or ATP (100 µM for 2 hours). *P<0.05, **P<0.01 and ****P<0.0001 (one-way ANOVA, Sidak's multiple comparisons test), n=8 biological replicates.

Figure S10

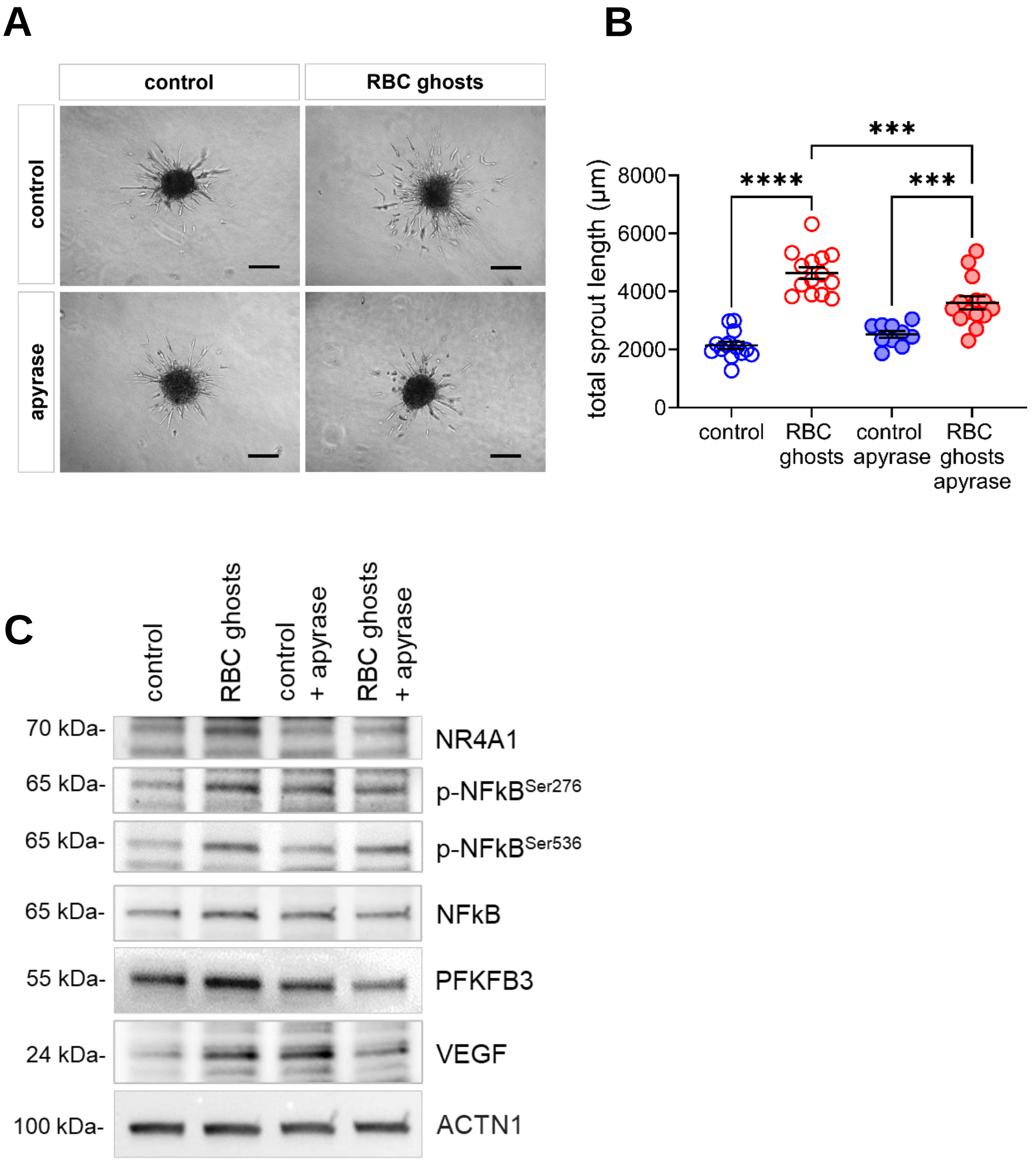

Figure S10. RBC membrane ATP as causal agent.

A, B Spheroid angiogenesis assay. Effects of ATP degradation using apyrase (5 U/mL for 30 min at 30°C) on endothelial tube formation, alone or after incubation with RBC ghosts. Representative brightfield images (A) and quantification of the effects of RBC ghosts (B) on endothelial tube elongation. (n=3 biologically independent RBC samples with multiple images analyzed per sample, per condition. Each dot represent single spheroid) Scale bars, 200 µm. **P<0.001 and ****P<0.0001 (one-way ANOVA, Sidak‘s multiple comparisons test).

D Immunoblot analysis of changes in NFκB p65, NR4A1, PFKFB3 and VEGF protein expression and NFκB p65 Ser276 or Ser 536 phosphorylation in endothelial cells incubated with RBC ghosts, alone or after pretreatment with apyrase (5 U/mlL for 30 min at 30°C) to degrade extracellular ATP.

Figure S11

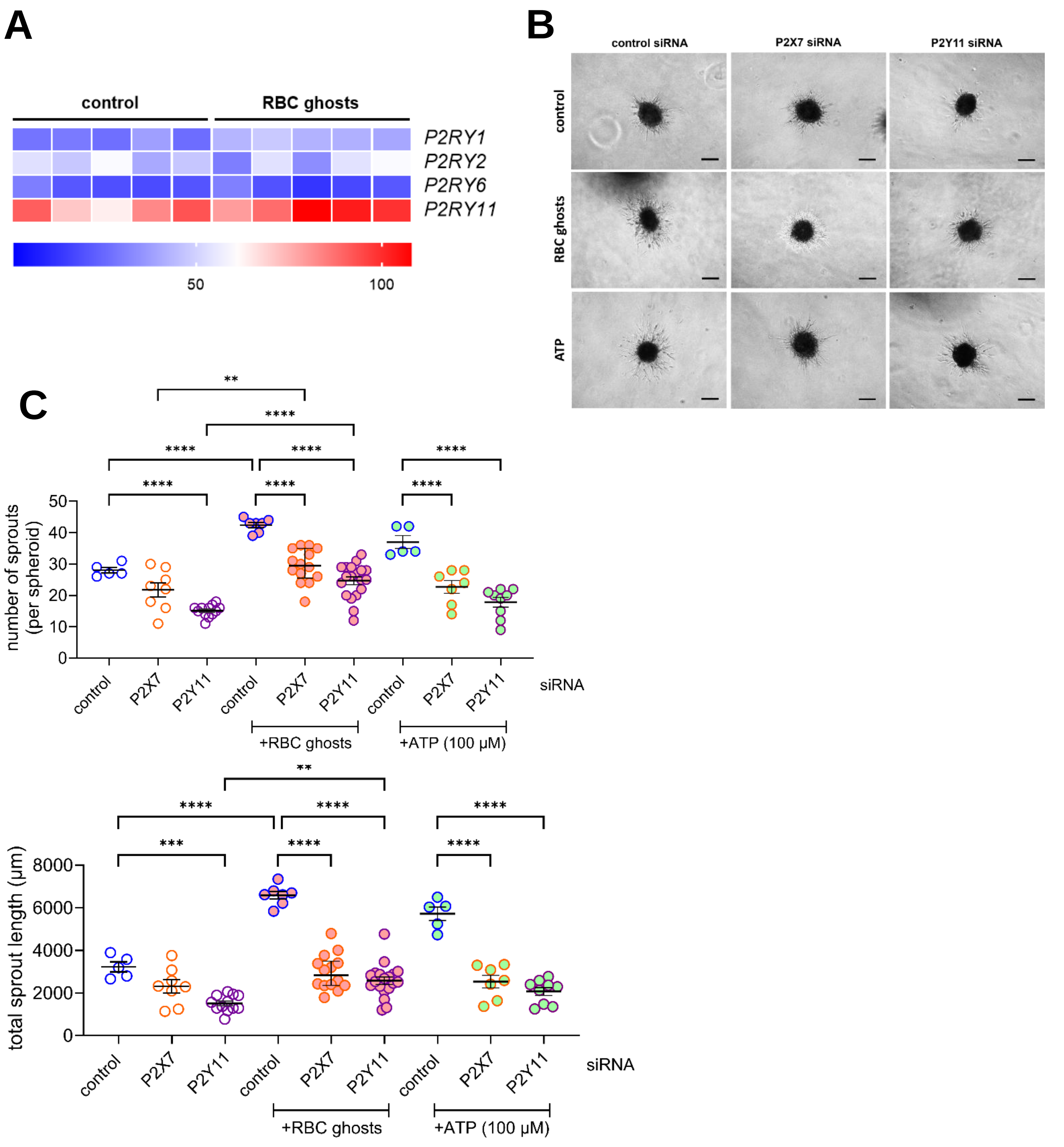

Figure S11. RNAi-mediated deletion of P2X7 or P2Y11 receptors abolishes endothelial angiogenic sprout formation in response to RBC ghosts or ATP.

A Heat-map visualizing mRNA transcript levels of metabotropic P2Y receptors in ECs, control- and RBC ghost-treated for 2 hours, based on RNA-seq experiments. Colors represent row-scaled normalized expression (Z-scores), with red and blue indicating relatively higher and lower expression, respectively.

B-D Spheroid angiogenesis assay in endothelial cells, 48 hours after transfection with P2X7R or P2Y11R or control siRNA, RBC ghost or ATP (100 µM) treated. (n=2 biologically independent RBC samples with multiple images analyzed per sample, per condition. Each dot represents one spheroid). Scale bars, 200 µm. **P<0.01, ***P<0.001 and ****P<0.0001 (two-way ANOVA, Sidak's multiple comparisons test in B and C.

Figure S12

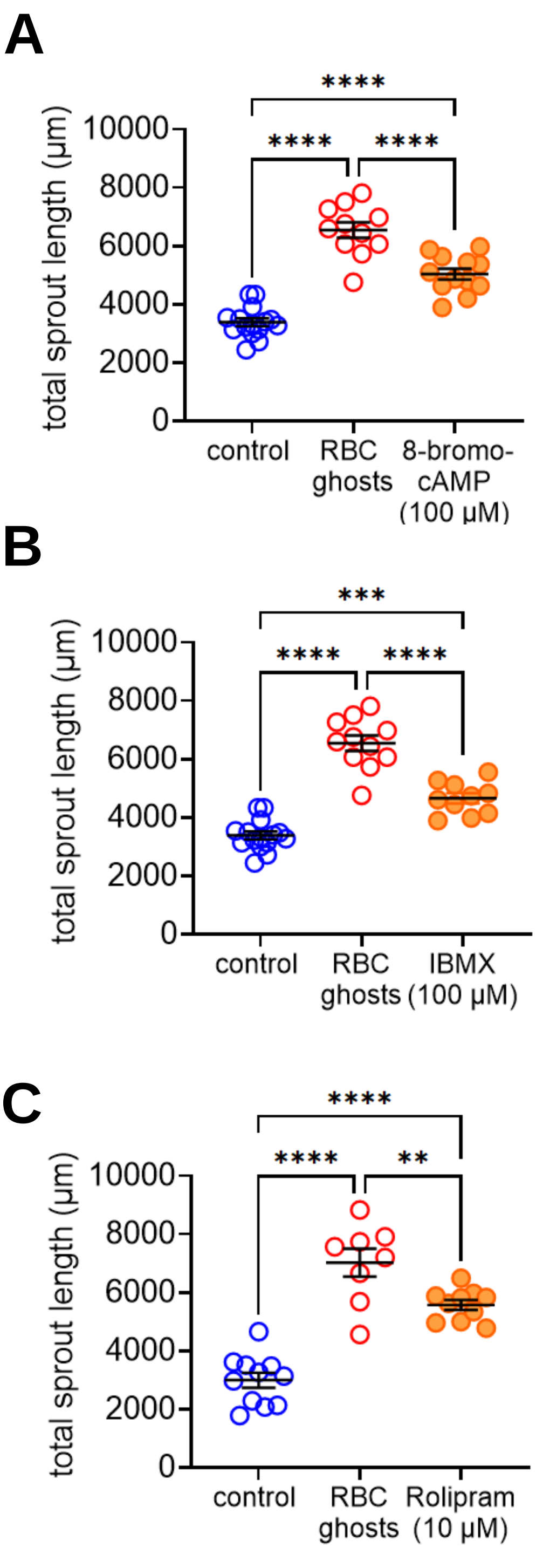

Figure S12. Pharmacologically increasing cAMP levels in endothelial cells phenocopies angiogenic effects of lysed erythrocyte membranes.

A-C Spheroid angiogenesis assay. Effects of the cell-permeable cAMP analogue 8-bromo-cAMP (A**;** 100 µM) or inhibitors of intracellular cAMP degradation, like the general phosphodiesterase inhibitor IBMX (**B**; 100 µM) or the specific inhibitor of endothelial cAMP PDE4 rolipram (C; 10 µM) on endothelial spout elongation, alone or after incubation with RBC ghosts. Note that 8-bromo-cAMP (B) and IBMX (C) were examined in the same experiment and share the same 'control' and 'RBC ghosts' group. **P<0.01, ***P<0.001 and ****P<0.0001 (one-way ANOVA, Sidak's multiple comparisons test). (n=3 independent biological replicates (RBC donors) analysed multiple images per sample, per condition. Each dot represents one spheroid)

Figure S13

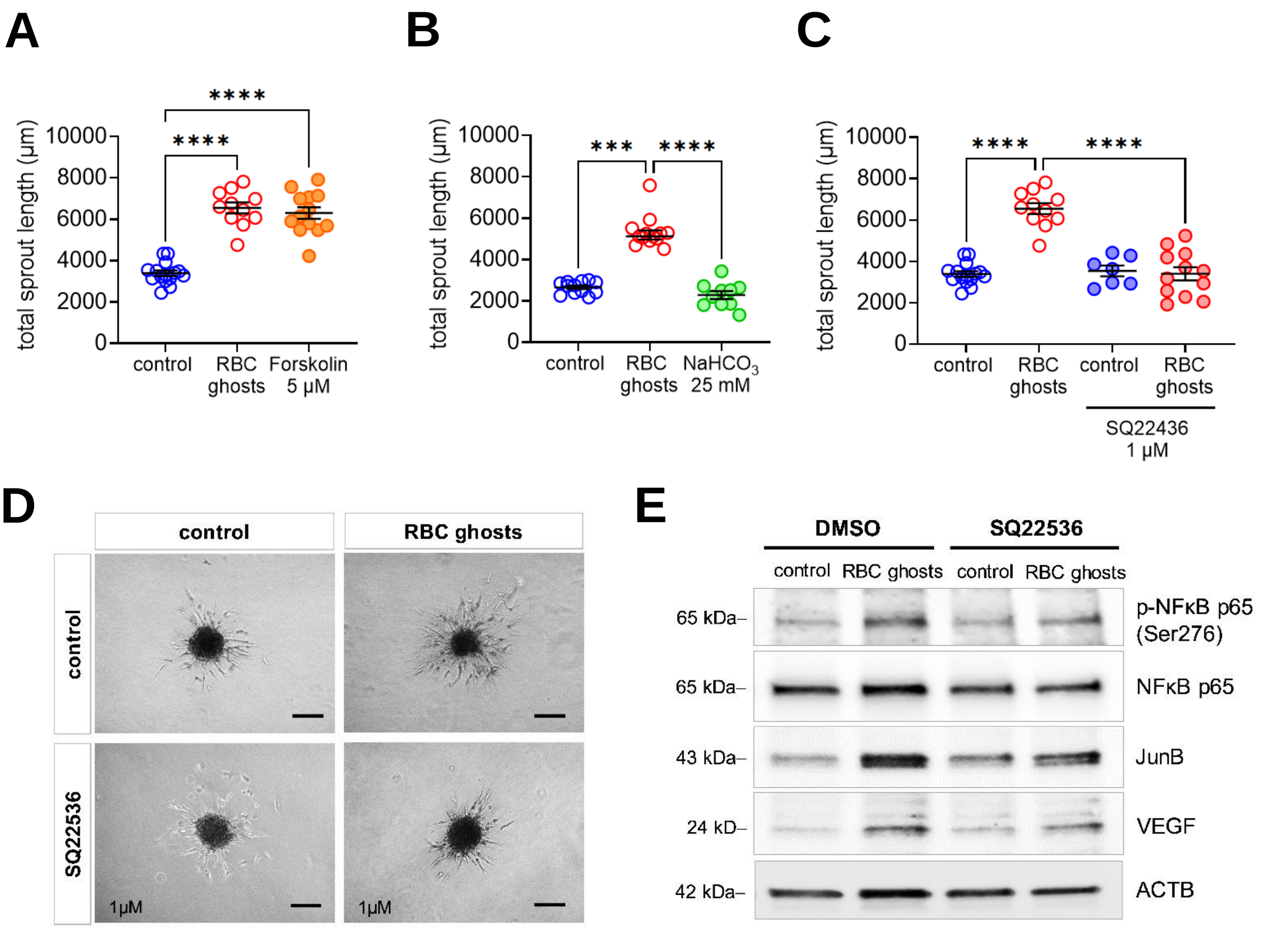

Figure S13. Pharmacologically increasing cAMP levels in endothelial cells phenocopies angiogenic effects of lysed erythrocyte membranes.

A-D Spheroid angiogenesis assay to examine the effect of membrane-bound or soluble adenylyl cyclase (AC) activation using Forskolin (5 µM; A) or $NaHCO_{3-}$ (25 mM; B) and membrane-bound AC inhibition using SQ22436 (1 µM; C, D) on endothelial sprout formation. Scale bars, 200 µm. ***P<0.001 and ****P<0.0001 (one-way ANOVA, Sidak's multiple comparisons test in A and C; Krukall-Wallis, Dunn's multiple comparisons test in B). (n=2 biologically independent RBC donors with multiple images collected per sample, per condition, Each dot represents one spheroid)

E Representative immunoblot membrane showing the effect of SQ22436 on NFκB p65 Ser276 phosphorylation and JUNB and VEGF protein expression in ECs, control or RBC ghost treated for two hours.
