# Supplementary material for: Lysed erythrocyte membranes metabolically prime endothelial cells for angiogenesis
Source: Angiogenesis. 2026 Sep 25;29(4):74. doi: 10.1007/s10456-026-10096-y (PMC13615041; doi:10.1007/s10456-026-10096-y)
Supplement: Supplementary file 2 [file 10456_2026_10096_MOESM2_ESM.docx]

| Gene | Symbol | Primer sequence (in 5’ − 3’ direction) |
| --- | --- | --- |
| C-X-C Motif Chemokine Ligand 8 | *CXCL8* | for− GACCACACTGCGCCAACAC  rev− CTTCTCCACAACCCTCTGCAC |
| Glucose-6-Phosphate dehydrogenase | *G6PD* | for− GTCCCACCAACTCTGCACTC  rev− ATGACACAAGGGGACCCAGG |
| Glucose Transporter-1 | *GLUT1* | for− GGCCAAGAGTGTGCTAAAGAA  rev− GGCCAAGAGTGTGCTAAAGAA |
| Hexokinase 1 | *HK1* | for− GCTCTCCGATGAAACTCTCATAG  rev− GGACCTTACGAATGTTGGCAA |
| Hexokinase 2 | *HK2* | for− GGAAAGGGGCAGAGGAACTC  rev− CCAAAGCACACGGAAGTTGG |
| Hypoxanthine Phosphoribosyl-transferase 1 | *HPRT1* | for− GTAATTGGTGGAGATGATCTCTCAACT  rev− TGTTTTGCCAGTGTCAATTATATCTTC |
| 6-Phosphofructo-2-Kinase/Fructose-2,6-Biphosphatase 3 | *PFKFB3* | for− CTCGCATCAACAGCTTTGAGG  rev− CAGTGTTTCCTGGAGGAGTC |
| Prostaglandin-endoperoxide synthase 2 | *PTGS2* | for− TTCAAATGAGATTGTGGGAAAATTGCT  rev− AGATCATCTCTGCCTGAGTATCTT |
| Tumor Necrosis Factor-Alpha | *TNFA* | for− TGAAAGCATGATCCGGGACG  rev− GTCACTCGGGGTTCGAGAAG |
| Vascular Endothelial Growth Factor A | *VEGFA* | for− GCTACTGCCATCCAATCGAG  rev− CTCTCCTATGTGCTGGCCTT |

**Table S1.** List of nucleotide primer sequences used for qPCR analysis of human genes.
